# Extracellular non-coding RNA signatures of the metacestode stage of *Echinococcus multilocularis*

**DOI:** 10.1371/journal.pntd.0008890

**Published:** 2020-11-30

**Authors:** María Eugenia Ancarola, Gabriel Lichtenstein, Johannes Herbig, Nancy Holroyd, Mara Mariconti, Enrico Brunetti, Matthew Berriman, Krystyna Albrecht, Antonio Marcilla, Mara Cecilia Rosenzvit, Laura Kamenetzky, Klaus Brehm, Marcela Cucher

**Affiliations:** 1 Departament of Microbiology, School of Medicine, University of Buenos Aires, Buenos Aires, Argentina; 2 Institute of Research on Microbiology and Medical Parasitology (IMPaM, UBA-CONICET), University of Buenos Aires, Buenos Aires, Argentina; 3 Department of Functional Materials in Medicine and Dentistry and Bavarian Polymer Institute, University of Würzburg, Würzburg, Germany; 4 Wellcome Sanger Institute, Wellcome Trust Genome Campus, Hinxton, Cambridge, United Kingdom; 5 Unit of Infectious and Tropical Diseases, San Matteo Hospital Foundation, Pavia, Italy; 6 Department of Clinical—Surgical, Diagnostic and Pediatric Sciences, University of Pavia, Pavia, Italy; 7 Departament de Farmàcia i Tecnologia Farmacéutica i Parasitologia, Universitat de València, València, Spain; 8 Joint Unit on Endocrinology, Nutrition and Clinical Dietetics, Instituto de Investigación Sanitaria-La Fe Valencia, València, Spain; 9 Institute for Hygiene and Microbiology, University of Würzburg, Würzburg, Germany; James Cook University, AUSTRALIA

## Abstract

Extracellular RNAs (ex-RNAs) are secreted by cells through different means that may involve association with proteins, lipoproteins or extracellular vesicles (EV). In the context of parasitism, ex-RNAs represent new and exciting communication intermediaries with promising potential as novel biomarkers. In the last years, it was shown that helminth parasites secrete ex-RNAs, however, most work mainly focused on RNA secretion mediated by EV. Ex-RNA study is of special interest in those helminth infections that still lack biomarkers for early and/or follow-up diagnosis, such as echinococcosis, a neglected zoonotic disease caused by cestodes of the genus *Echinococcus*. In this work, we have characterised the ex-RNA profile secreted by *in vitro* grown metacestodes of *Echinococcus multilocularis*, the casuative agent of alveolar echinococcosis.

We have used high throughput RNA-sequencing together with RT-qPCR to characterise the ex-RNA profile secreted towards the extra- and intra-parasite milieus in EV-enriched and EV-depleted fractions. We show that a polarized secretion of small RNAs takes place, with microRNAs mainly secreted to the extra-parasite milieu and rRNA- and tRNA-derived sequences mostly secreted to the intra-parasite milieu. In addition, we show by nanoparticle tracking analyses that viable metacestodes secrete EV mainly into the metacestode inner vesicular fluid (MVF); however, the number of nanoparticles in culture medium and MVF increases > 10-fold when metacestodes show signs of tegument impairment. Interestingly, we confirm the presence of host miRNAs in the intra-parasite milieu, implying their internalization and transport through the tegument towards the MVF. Finally, our assessment of the detection of *Echinococcus* miRNAs in patient samples by RT-qPCR yielded negative results suggesting the tested miRNAs may not be good biomarkers for this disease.

A comprehensive study of the secretion mechanisms throughout the life cycle of these parasites will help to understand parasite interaction with the host and also, improve current diagnostic tools.

## Author summary

Extracellular RNAs (ex-RNAs) are secreted by cells through association with proteins or extracellular vesicles (EV). In the context of parasitism, ex-RNAs represent novel communication intermediaries with promising potential as biomarkers. In order to better understand the role ex-RNAs may play in the context of the zoonotic disease echinococcosis, we have characterised the RNA profile secreted by the larval stage (metacestode) of *Echinococcus multilocularis*.

By analysing the products secreted towards the extra- and intra-parasite milieus, we demonstrate that the metacestode displays a polarized secretion of different classes of small non-coding RNAs (sRNAs). In addition, we show that EV secretion occurs mainly towards the inner fluid of the metacestodes. Interestingly, we confirm the presence of host sRNAs in the intra-parasite milieu, implying their internalization and transport through the tegument. Finally, the detection of *Echinococcus* miRNAs in patient samples yielded negative results suggesting the tested miRNAs may not be good biomarkers for this disease.

In summary, our results provide a detailed description of the ex-RNA landscape of the *E*. *multilocularis* metacestode together with information on the distribution of the detected RNA classes in different extracellular compartments. This information is of importance to better understand host-parasite interaction and also, to improve current diagnostic tools.

## Introduction

Cell-free or extracellular RNAs (ex-RNAs) released by animal cells were first described in the 70´s, based on *in vitro* experiments, and subsequently found circulating in plasma *in vivo* [1–3]. Nowadays, it is known that ex-RNA stability in RNAse rich environments can be conferred by association with proteins or lipoproteins [4–7] or by packaging in extracellular vesicles (EV) [8]. Recently, it was proposed that the acquisition of a protective secondary conformation could also stabilize certain ex-RNAs [9]. One abundantly secreted class of RNA is small non-coding RNAs (sRNAs). This term groups several classes of regulatory RNAs that display sizes shorter than 200 nt such as microRNAs (miRNAs) and sRNAs derived from tRNAs. Both are able to exert a regulatory action on gene expression [10,11] and thus their role in intercellular communication is being thoroughly investigated.

In the context of parasitism, ex-RNAs represent new and exciting communication intermediaries not only between individuals of the same species, but also between parasites and hosts. With respect to helminth infections, the potential of ex-RNA for use in novel diagnostic tools is highly promising [12,13] mostly in those diseases that still lack biomarkers for early and/or follow-up diagnosis, such as echinococcosis [14]. Human echinococcosis is a zoonosis produced by the development and growth of the metacestode stage of cestodes of the genus *Echinococcus*. The adult stage of these tapeworms grows in the small intestine of carnivores. The two most clinically relevant forms of this disease are alveolar (AE) and cystic (CE) echinococcosis, caused by *Echinococcus multilocularis* and *Echinococcus granulosus sensu lato* (s.l.), respectively. In CE, the metacestode develops as cystic lesions filled with an inner fluid (hydatid fluid), mainly in liver and lung, while in AE it develops as a multivesicular budding mass that infiltrates host tissue (mostly liver) and gradually expands during decades eventually spreading to other organs [15]. Additionally, in late infections the *E*. *multilocularis* metacestode can progressively become necrotic in the central region of the parasite tissue [16] and may consequently release intracellular molecules and vesicles into the extra-parasite environment.The metacestode stage of these parasites consists of a fluid-filled vesicle lined by an inner cellular layer (germinal layer) and an outer acellular layer (laminated layer), unique among cestodes. The laminated layer is a specialized extracellular matrix synthesized by the tegument, the outermost syncytial stratum of the germinal layer [17].

In human CE and AE, parasite development takes years until clinical signs and symptoms arise, and diagnosis is based on clinical findings, epidemiological data, imaging techniques and serology [18]. However, non-invasive biomarkers to detect the parasites at an early stage of disease or to accurately determine parasite viability (for drug treatment follow-up or recurrence assessment) are still lacking.

Since the first report on RNA content in EV from parasitic helminths [19], several others have shown that nematode, trematode and cestode parasites secrete ex-RNAs [20]. In nematodes and trematodes, sRNAs have been reported to be present in both EV-enriched and EV-depleted fractions obtained from parasite conditioned medium [21–23]. The term EV refers to a wide variety of membrane bound structures that contain proteins, lipids and nucleic acids and are released by cells. In the case of cestodes, we have previously demonstrated that the metacestode stages of *Taenia crassiceps* and *Mesocestoides corti* secrete EV with sRNAs *in vitro* [24]. In that work, we also observed that the metacestode stage of *E*. *multilocularis* can hardly secrete EV to the extra-parasite medium, probably due to the presence of the laminated layer. Other authors have reported the presence of EV in the hydatid fluid of *E*. *granulosus* s.l. metacestodes [25–28] and the culture medium of *Echinococcus* spp. protoscoleces and/or metacestodes [27–29]. Recently, it was reported that EV from *E*. *multilocularis* contain sRNAs [30]. However, no quantitative analyses of EV secretion to the extra-parasite milieu or the profiling of ex-RNAs in the non-vesicular fraction of the secretion poducts of these parasites has been performed to date.

In this work, using conventional protocols for EV and ribonucleoprotein complex enrichment, we aimed to identify the main RNA classes secreted *in vitro* by the metacestode stage of *E*. *multilocularis* to both the intra- and extra-parasite milieus to evaluate the potential of ex-RNAs as novel echinococcosis biomarkers.

## Materials and methods

### Ethics statement

Animal experiments were carried out in accordance with European and German regulations on the protection of animals (Tierschutzgesetz) and were approved by the government of Lower Franconia under permit no. 55.2–2531.01-61/13.

The study involving human samples complies with the Declaration of Helsinki. In the case of AE samples, the procedure has been approved by the local ethics comission of the Faculty of Medicine of the University of Würzburg (https://www.med.uni-wuerzburg.de/ethik-kommission). Data privacy protection was guaranteed by anonymization of serum samples. In the case of CE samples, patients signed a consent form (approved protocol N° 20180060669, Direzione Scientifica, Comitato Etico Area Pavia).

### Parasites

*Echinococcus multilocularis* metacestodes from isolates H95, J2012, Ingrid, GH09 and RD15 were maintained by serial intraperitoneal passage in *Meriones unguiculatus* as previously described [31] at the animal facilities of the Institute of Hygiene and Microbiology, University of Würzburg.

*In vitro* generated metacestodes were obtained in co-culture with rat hepatocytes as previously described [31].

### Parasite *in vitro* culture

Metacestode culture was performed as already described [24] with minor modifications. Briefly, 30–60 ml of parasites (mean diameter 0.61 cm ± 0.12) were incubated in serum-free medium to a final volume of 150 ml and the axenization step was conducted for 1 day. Cultures were classified into *Active* (Viable) and *Transitional* according to parasite tegument integrity. For this, metacestodes from each culture were photographed, counted and classified into non-stained and phenol red stained. Cultures with ≤ 6% of phenol-red stained parasites were classified as Active, and cultures with ≥ 15% stained parasites were considered Transitional. To check if phenol red could be used as a tegument integrity indicator, its staining efficiency was compared to vital staining of metacestodes with eosin (final concentration = 0.02%) (S1A Fig). Only turgent metacestodes were used, collapsed metacestodes and/or with detached germinal layer were removed from the cultures before axenization or incubation (S1B Fig).

### Collection of extracellular samples

Parasite conditioned serum-free media were collected and centrifuged according to [32] but with modifications. Briefly, culture medium was centrifuged for 20 min at 2,000 x g at 4°C and 30 min at 10,000 x g at 4°C. The obtained supernatant was ultracentrifuged for 70 min at 100,000 x g and 4°C in a Sorvall WX+ Ultracentrifuge (Thermo Scientific) with rotor TH-641, washed with PBS and ultracentrifuged again. The EV-enriched pellet (P100 fraction) was resuspended in 100 μl sterile Hank´s solution (Thermo Scientific, Germany) for further use.

Metacestode inner vesicular fluid (MVF) was collected by individual sterile puncture of *in vitro* grown metacestodes with a 26 G syringe and processed as described above. A few microliters of liquid were discarded before dispensing the fluid in the collection tube to avoid carry over of cyst wall content that might be present in the needle.

The EV-depleted supernatants (S100 fraction) of the ultracentrifugation step were concentrated with 3-kDa Amicon Centrifuge Filter (Merck Millipore) devices at 5000 x g at 4°C using a fixed angle rotor, followed by 2 washes with PBS [21].

**Fig 1:**
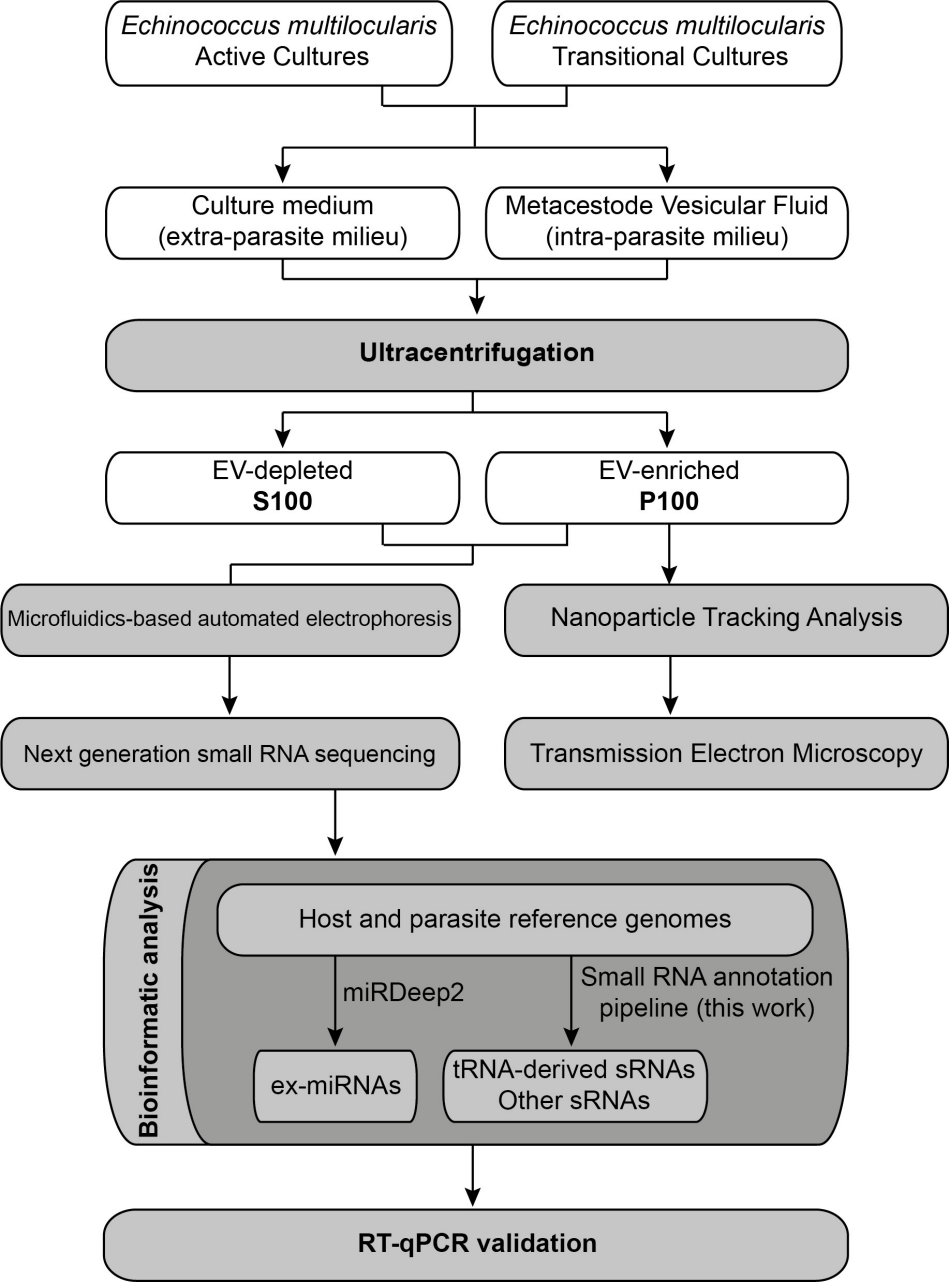
Workflow used for ex-RNA identification in *E*. *multilocularis* metacestodes.

The different EV-enriched and EV-depleted fractions obtained after ultracentrifugation will be called P100 and S100, respectively, in order to avoid mechanistic definitions since the methodology used cannot achieve absolute purification [33]. A diagram of the experimental design of this work is shown in Fig 1.

### Nanoparticle tracking analysis (NTA)

Nanoparticle concentration and size distribution of P100 fractions were measured in a NanoSight NS 500 Nanotracker (Malvern Instruments). Samples were stored for a maximum of 10 days at 4°C until measurement. Samples of active metacestode culture medium were diluted to 1/100 and from transitional metacestodes to 1/100 or 1/1000. MVF was diluted to 1/100 and 1/1000 for active and transitional metacestodes, respectively. Dilutions were done in particle-free sterile distilled water immediately prior to measuring. Ten 60-second recordings were performed on each sample, and the three technical replicates with highest values were averaged and used for analysis. The parameters used for detection were: threshold = 10; camera level = 16 and temperature = 23.3°C. The NTA software (Version 2.3) was used. Results were normalized to the initial volume of metacestodes or MVF used for each experiment using the formula: Normalized N° np/ml = N° np/ml x DF x V_i P100_ x 1/V _Met or MVF_, where np = nanoparticles, DF: dilution factor, V_i P100_ = initial volume (ml) in which P100 was resuspended, V _Met or MVF_: Volume (ml) of metacestodes (Met) or metacestode vesicular fluid (MVF).

### Transmission electron microscopy (TEM)

Aliquots of P100 fractions were resuspended in PBS, fixed in Karnovsky’s fixative (0.5% glutaraldehyde, 2.5% paraformaldehyde) and were negatively stained with 2% uranyl acetate in double destilled water on a membrane acrylic–coated grid at Laboratorio Nacional de Investigación y Servicios de Microscopía Electrónica (LANAIS-MIE), School of Medicine, University of Buenos Aires.

### Small RNA library construction and sequencing

For P100 sRNA library construction, RNA from the following volumes of pools of samples was obtained: 500 ml (7 samples) and 200 ml (3 samples) from active and transitional culture media, respectively; 30 ml (6 samples) of MVF from active cultures; 13 ml (3 samples) and 10 ml (5 samples) of MVF from non-stained and stained metacestodes, respectively, from transitional cultures. For S100 sRNA library construction, RNA was isolated from 175 ml (2 samples) and 190 ml (2 samples) from active and transitional culture media, respectively; 44 ml (7 samples) of MVF from active cultures; and 16 ml (3 samples) and 9 ml (4 samples) of MVF from non-stained and stained metacestodes, respectively, from transitional cultures.

RNA was isolated with Trizol LS (Life Technologies, Germany) and the aqueous phase obtained was enriched in < 200-nt RNA or > 200-nt RNA with mirVana miRNA Isolation Kit (Life Technologies, Germany). In both cases, RNA was eluted twice by incubating 10 minutes with 100 μl of pre-warmed nuclease-free water at 90°C and then precipited as already described for 2 h at -80°C and then O.N. at -20°C [24].

The RNA concentration and size in each sample were analysed with an Agilent 2100 Bioanalyzer (Agilent Technologies, U.S.A). Small RNA chips and RNA 6000 pico chips were used for sRNA (< 200 nt) and large RNA analysis (> 200 nt), respectively.

After checking size profile distribution, 1.8 to 200 ng of RNA were used for library construction with the NEBNext Multiplex Small RNA Library Prep Set for Illumina (New England Biolabs, U.S.A). To recover all the ligated RNAs present in each sample, size selection of the obtained cDNA was performed with Agencourt AMPure XP Beads (Beckman Coulter, U.S.A.) according to the manufacturer´s instructions. Sequencing was performed on a HiSeq 2500, v4 (Illumina, 125 bp pair-end platform). Quality control, library preparation and sequencing were performed at the Wellcome Sanger Institute, Cambridgeshire, United Kingdom.

Raw reads were deposited at the European Nucleotide Archive (https://www.ebi.ac.uk/ena) under the accession number ERP121841.

### RNA-sequencing data analysis

*Data preprocessing*: Fastqc (version 0.11.7, https://www.bioinformatics.babraham.ac.uk/projects/fastqc/) quality control was executed with default parameters on all libraries. Reads with ≥ 18-bp length and per base sequence quality mean PHRED ≥ 20 were retained. A three-step approach was implemented by in-house bash scripts (https://github.com/MCucher/Emul_exRNA_scripts_and_data.git) to: i) remove sequencing adaptors via cutadapt (version 1.18,—error-rate = 0.3—trim-n–a NEBnext_E7300.fasta [34]; ii) re-pair disordered reads using repair.sh from the JGI BBTools package with default parameters (version 37.10, https://sourceforge.net/projects/bbmap/), each paired and singleton read were concatenated in a single file per library and overlapping paired-end reads were merged with the JGI BBmerge algorithm (version 37.10, minoverlap0 = 8 minoverlap = 12 mininsert = 18 mininsert0 = 17, [35]) and lastly iii) remove low-quality bases using Trimmomatic SE sliding window approach (version 0.36, SLIDINGWINDOW:4:20 MINLEN:18 [36]).

*Identification of miRNAs*: miRNAs from *E*. *multilocularis* and *Mus musculus* were identified with mirDeep2 [37] as already described with some modifications [38]. Sequences were mapped to either reference genome with the read aligner Bowtie (mapper module) using default parameters, allowing only alignments with 0 mismatches in the first 18 nt of a read sequence and up to two mismatches after nt 18, and keeping only reads that did not map more than five times to the genome. *Mus musculus* was used as reference since the repertoires of miRNAs and non-coding RNAs are better characterized than in rat. Briefly, for parasite miRNA identification the *E*. *multilocularis* genome assembly version 4 downloaded from the Sanger Institute FTP site [ftp://ftp.sanger.ac.uk/pub/project/pathogens/Echinococcus] and *E*. *multilocularis* mature and precursor miRNAs from [39,40] and metazoan mature miRNAs from miRBase (version 21) were used as input data together with the high quality reads obtained for each library after preprocessing. Only those sequences that fulfilled the following criteria were considered miRNAs and hence included in the analyses: miRDeep2 score ≥ 4; significant randfold p-value “yes”; no rfam alert; no sequence identity with non-coding RNA (excluding miRNAs) present in RNA central database and read count number ≥ 350 for at least one of the sequences that aligns with the respective precursor. The miRDeep score reflects the probability that a precursor corresponds to a genuine miRNA [41]. The value of this score was selected in order to obtain the lowest percentage of estimated false positive novel miRNAs and the highest percentage of detected known miRNAs as previously reported [40]. For vertebrate miRNA identification, the input files corresponded to the *M*. *musculus* genome assembly GRCm38.p6 downloaded from the Ensembl FTP site [ftp://ftp.ensembl.org/pub/release-95/fasta/mus_musculus/dna/] and *M*. *musculus* mature and precursor miRNAs and metazoan mature miRNAs downloaded from miRBase (version 21).

When miRNAs with high identity between *E*. *multilocularis* and *M*. *musculus* were identified (≤ 1 SNP), the origin of the miRNA was established by taking into consideration: i) the pre-miRNA sequence to which the highest number of reads could be mapped and ii) the absence of mismatches to the pre-miRNA in the most frequent read sequence. When the terminal 3´nucleotide corresponded to a non-templated U it was not considered as a mismatch but as a non-templated added nucleotide [40].

*Identification of other sRNAs*: High quality reads were also aligned to the *E*. *multilocularis* (v4) and *M*. *musculus* (GRCm38) genomes using Bowtie (version 1.2.1.1, bowtie-align—wrapper basic-0 -v3—sam—best—time—threads 8, [42]) to obtain a SAM file. For annotation of other sRNAs, first a sequence identity analysis was performed using BLAST (version 2.7.1, -task = blastn-short -outfmt = 6 -max_target_seqs = 1 -evalue = 0.01 -num_threads = 8, [43]). These files were used to query a set of two different *ad hoc* reference databases containing: i) *E*. *multilocularis*: miRNA and pre-miRNA sequences from [39,40] and non-coding RNAs downloaded from RNA central database (http://rnacentral.org/) and ii) *M*. *musculus*: miRNA and pre-miRNA sequences from miRBase, tRNAs from GtRNAdb (http://gtrnadb.ucsc.edu/genomes/eukaryota/Mmusc10/Mmusc10-summary.html) and those non-coding RNA sequences classified as “Other” in RNA central database excluding miRNAs and tRNAs. tRNA sequence annotation was performed with tRNAscan-SE [44]. In-house databases are available at https://github.com/MCucher/Emul_exRNA_scripts_and_data.git. The final step was to parse the tabular blastn-short tables to excel and only reads that fulfilled the following criteria were retained: gap = 0, hit start 1 or 2, mismatch = 0, read coverage ≥ 95 and read count number ≥ 350. Those sequences that passed these filters and were present in *E*. *multilocularis* and *M*. *musculus* datasets from the same library were individually analysed to determine their origin. This was established by i) determining the percentage of coverage over sequence length and ii) identifying the number of mapping reads per locus. The final selection was made on highest coverage and highest number of mapping reads to each genome. When the origin of the sequence could not be confidently determined by these criteria, sequences were termed Ambiguous and were not used for further analyses. Otherwise, they were termed Unambiguous and used to classify the different types of RNAs detected in each sample.

For generating an abundance ranking specific for each sRNA-type and in order to compare sRNA proportions among samples, every sRNA sequence was normalized with respect to the total number of counts associated to the corresponding sRNA biotype (miRNA, sequences derived from tRNA, rRNA and SRP) in a given sample [38,40]. The sequence of the most frequent read is reported.

All bioinformatics analyses were performed in a local server at IMPaM, which is part of Sistema Nacional de Computación de Alto Desempeño (SNCAD), ID 924 Ministerio de Ciencia, Tecnología e Innovación Productiva (MINCyT), Argentina.

### Plasma and sera from patients

*Echinococcus multilocularis* samples: Samples from patients with presence of liver lesion detected by ultrasonography were defined positive if immunodiagnosis results were positive for *E*. *multilocularis*. Negative samples corresponded to patients with negative immunodiagnosis.

Serum (250 μl) from 3 alveolar echinococcosis patients taken before and after treatment (surgery and/or albendazole) and from 3 negative patients were obtained from the Serology Department of the Institute of Hygiene and Microbiology, University of Würzburg, Germany. Diagnosis was performed by HAT (antigens from *E*. *granulosus* s.l. (Fumouze)) and ELISA (EG55 antigen, i.e recombinant Ag B, from *E*. *granulosus* s.l. [45]; EM10 and total larva antigens from *E*. *multilocularis*). Samples were kept at -20°C until RNA isolation.

*Echinococcus granulosus* s. l. samples: Samples from patients with presence of CE liver cyst detected by ultrasonography were defined as positive, otherwise they were considered negative.

Serum and plasma (190 to 250 μl) collected in BD Vacutainer EDTA tubes were obtained from Policlinico San Matteo Hospital Foundation, Pavia, Italy. Cysts were classified according to the WHO-IWGE classification and patients were tested for routine diagnostic purposes in the laboratory of Parasitology of San Matteo Hospital Foundation, using ELISA (RIDASCREEN Echinococcus IgG, R-Biopharm, Darmstadt, Germany). An equal number of samples from each of the following groups were analysed: active (CE1-2), transitional (CE3b), inactive (CE4, CE5) and control group (negative patients with no parasitic cysts). All the CE enrolled patients have one single cyst localized in the liver. Samples were kept at -80°C with Trizol LS until RNA isolation.

### Reverse transcription (RT) and quantitative PCR (qPCR)

*Culture medium samples*: Individual culture medium or pool of samples were used, and RNA isolation was performed as described in section “Small RNA library construction and sequencing”. Transitional cultures contained 15–30% of phenol red-stained metacestodes. Only the < 200-nt RNA was purified. After precipitation and drying, RNA was resuspended in 20 μl of RNAse-free water.

cDNA synthesis was performed by stem-loop (miRNAs, tRNA^Glu^) or poly-A (tRNA^Ala^, tRNA^Gly^ and SRP) RT. Stem-loop (SL) RT was performed as follows: 5 μl of RNA were added to 2 μl of RNAse-free H_2_O and incubated 5 min at 94°C, followed by ≥ 1 min in ice. Then, the following reagents were added to reach the indicated final concentrations: reaction buffer 1X, DTT 5 mM, dNTPs 0.5 mM, SL primers 0.0125 μM each, RNAse OUT 0.4 U/μl, SuperScript 4 10U/μl, RNAse-free H_2_O to final volume 20 μl. Reactions were incubated 30 min at 16°C, 30 min at 50°C, 10 min at 80°C. Poly-A reaction was performed in a final volume of 10 μl with reaction buffer 1X, ATP 1 mM, E-PAP (New England Biolabs, U.S.A) 0.5 U/μl. Incubation was performed 1 h at 37°C followed by 10 min at 65°C. RT was carried out in 13 μl of reaction with 5 μl of poly-A RNA, poly-T adaptor 2.8 μM and 0.77 mM dNTPs. This was incubated 5 min at 65°C, followed by ≥ 1 min in ice. Then, reaction buffer 1X, DTT 5 mM, RNAse OUT 0.4 U/μl, SuperScript 4 10U/μl, RNAse-free H_2_O to final volume 20 μl were added and incubated 30 min at 50°C, followed by 10 min at 80°C. Stem loop primers and Poly T adaptor sequences are described in S1 Table.

The qPCR mix consisted of 1x HOT FIREPol EvaGreen qPCR Mix Plus (ROX) (Solis Biodyne) and 0.2 μM of each primer. Real time PCR was performed in a StepOne Plus cycler (Applied Biosystems). The cycling conditions were: 95°C 15 min, followed by 40 cycles of 95°C 15 sec, annealing temperature (detailed in S1 Table) 20 sec, 72°C 30 sec. Fluorescence data was collected at 72°C. No reverse transcriptase and no template reactions were also performed. Sequence, annealing temperature and efficiency of each PCR primer are described in S1 Table. PCR efficiencies were determined using LinRegPCR [46]. Amplification product specificity was assessed by melting curve analysis and gel electrophoresis. For miR-71-5p, a hydrolysis probe (5´- ACTGGATACGACTCTCACTA-3´) with 5´FAM and 3´end MBG modifications (Biomers, Germany) was also tested. In this case, Luna Universal Probe qPCR Master Mix (New England Biolabs, U.S.A) was used. The reaction was carried out in a final volume of 10 μl with 0.2 μM of each primer and 0.4 μM probe. Cycling conditions were 95°C 1 min, followed by 40 cycles of 95°C 15 sec, 60°C 30 sec. Fluorescence data was collected at 60°C.

Expression levels were calculated by the efficiency correction method [47] and were normalized to culture medium used as input. Cycle threshold (Ct) values were considered for calculations if ≥ 2.5 cycles with respect to no template or no transcriptase controls, whatever the least [33].

*MVF samples*: RNA from 17 ml, 19 ml and 45.5 ml of MVF from active cultures was isolated with Trizol LS. Stem-loop cDNA was synthesized as described above. For miR-122-5p qPCR detection, a hydrolysis probe (5´- ACTGGATACGACCAAACACC-3´) with 5´FAM and 3´end MBG modifications (Biomers, Germany) was used as described above.

*Plasma/sera samples*: RNA isolation, cDNA synthesis and qPCR were performed as described above. After precipitation and drying, RNA was resuspended in 20 μl of RNAse-free water. Five μl of RNA were used as input for the SL RT reaction. As positive amplification control, endogenous hsa-miR-423-5p was used (S1 Table). For miR-71-5p, a hydrolysis probe (5´- ACTGGATACGACTCTCACTA-3´) with 5´FAM and 3´end MBG modifications (Biomers, Germany) was also tested as described above.

### Statistical analysis

Nanoparticle concentrations and sizes were compared by one-tailed Mann-Whitney or Kruskal-Wallis tests. Relative expression levels of P100 and S100 fractions from each type of culture were compared by one-tailed Mann-Whitney test. P values were considered significant when ≤ 0.05.

Enrichment analysis of miRNAs between the S100 fraction of active culture medium and metacestode tissue was performed by linear regression [48]. Metacestode miRNA counts were retrieved from a previous work [40]. microRNAs with -1 ≤ log_2_ fold change ≤ 1 were considered equally abundant in both samples.

The software used for statistical analysis was GraphPad Prism version 5.01.

## Results

### The *Echinococcus multilocularis* metacestode secretes nanoparticles mainly to the intra-parasite milieu

**Fig 2:**
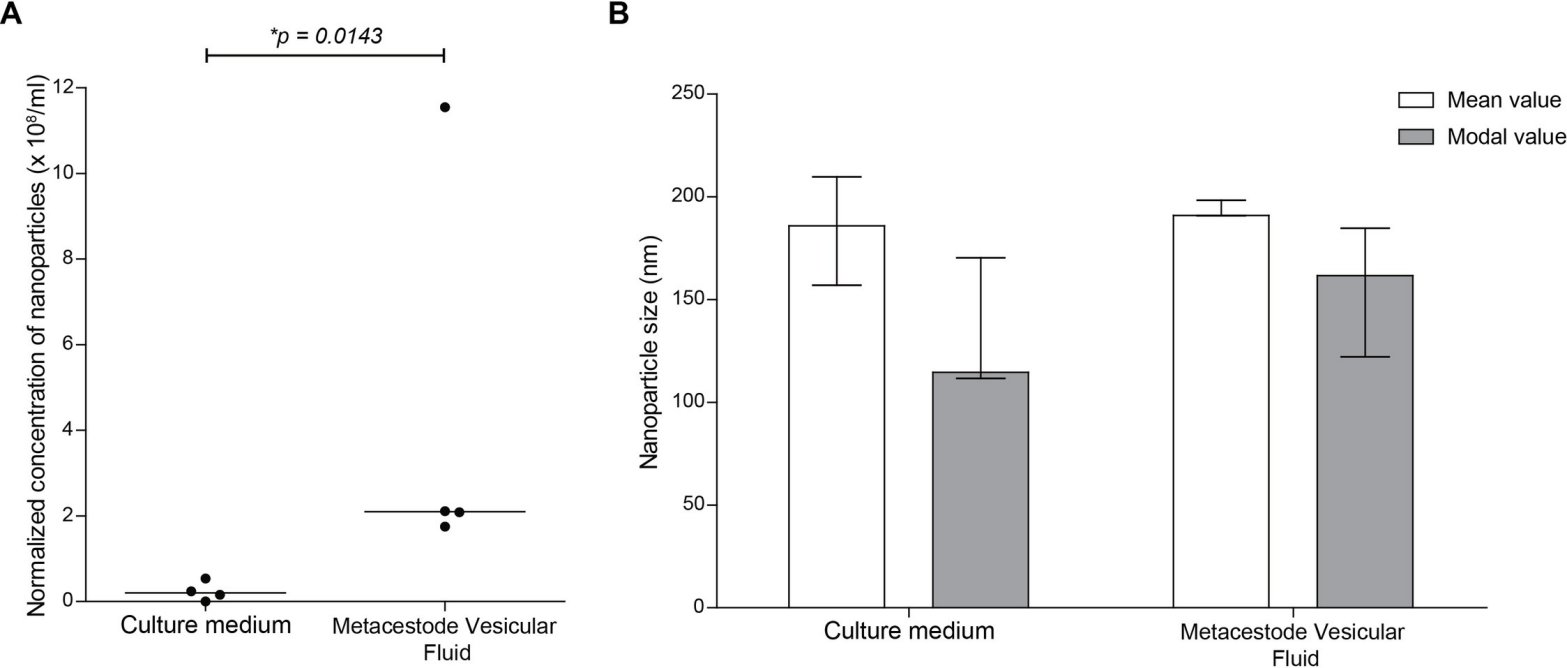
Nanoparticle tracking analysis of extra- (culture medium) and intra- (metacestodes vesicular fluid) parasite secretions of the *E*. *multilocularis* metacestode *in vitro*. A) Normalized concentration of nanoparticles expressed in number of nanoparticles/ml. Each dot represents a biological replicate and lines indicate the median values. B) Mean and modal size of the detected nanoparticles. Bars indicate median size with range of the corresponding value.

In order to characterise EV secretion towards the extra- and intra-parasite milieus in *E*. *multilocularis* metacestodes, we analysed the presence of nanoparticles in P100 (EV-enriched) from culture media and MVF from active cultures (Fig 1). Using NTA, we observed that a low concentration of nanoparticles could be detected in the culture media conditioned by metacestodes, while a significantly higher number of nanoparticles (10 to 20-fold) could be detected in MVF (p = 0.0143; Fig 2A). No significant differences were observed in the size of the detected nanoparticles, with mean sizes of 186 nm (157–210) and 191 nm (191–198) in culture medium and MVF, respectively, and modal sizes of 115 nm (112–170) and 162 nm (122–185), respectively (Fig 2B).

### Ex-RNAs from *Echinococcus multilocularis* mainly correspond to miRNAs and derived sequences from tRNAs and rRNAs

**Fig 3:**
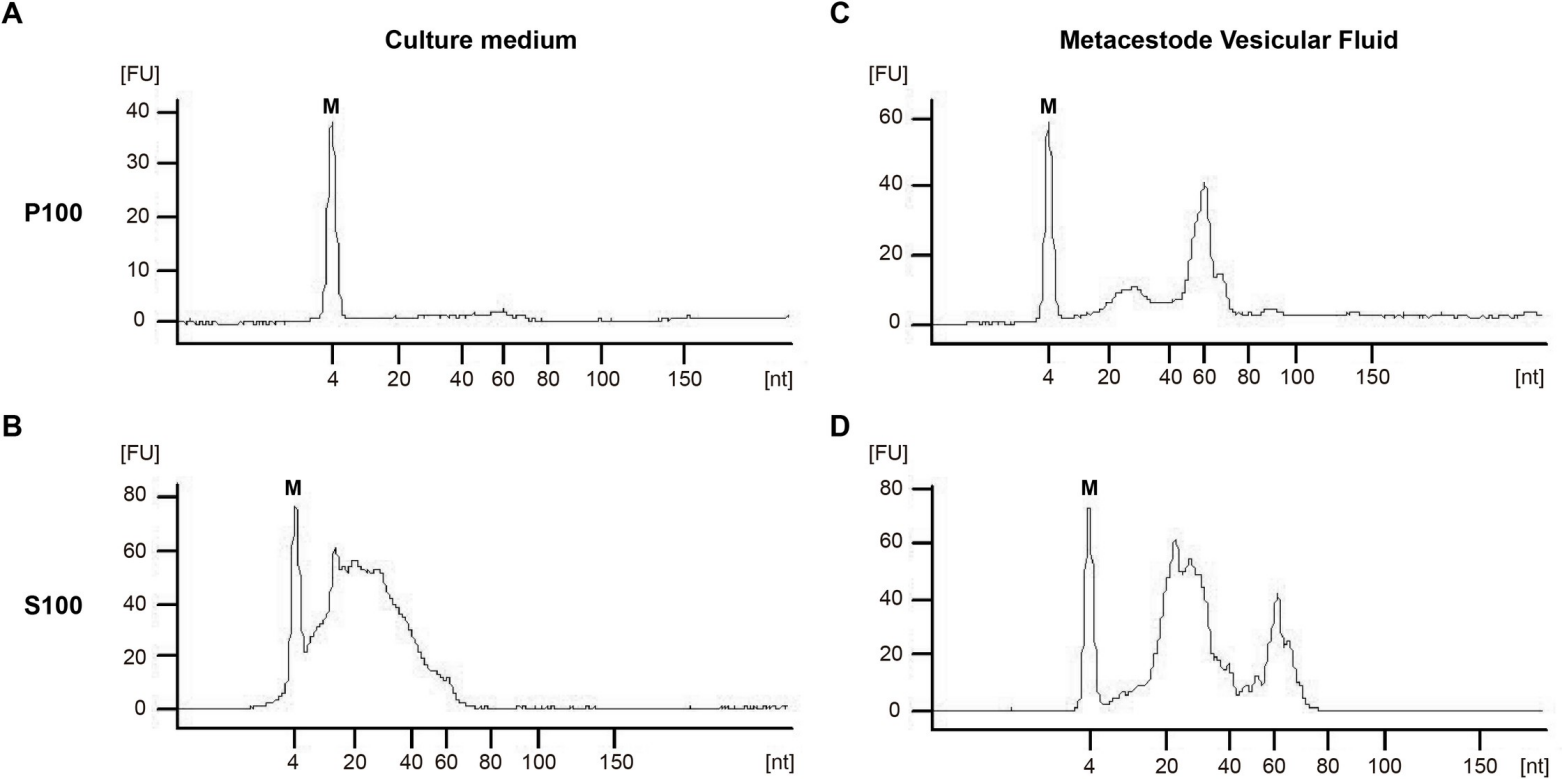
*Echinococcus multilocularis* extra- and intra-parasite secretions contain sRNAs. Analysis of the sRNA content (< 200 nt) present in the P100 and S100 fractions of culture medium (A, B) and metacestode vesicular fluid (C, D) of *E*. *multilocularis* active metacestodes. M: marker. FU: fluorescence units.

In order to analyse the presence and size distribution of RNA in the secretion products of active metacestodes, we analysed the P100 and S100 fractions of culture medium and MVF (Fig 1). We observed that in culture medium sRNAs could be detected only in the S100 fraction with peaks ranging between 20–40 nt (Fig 3A and 3B). In the case of MVF, both P100 and S100 samples contained sRNAs, with peaks between 20–40 nt and 60 nt (Fig 3C and 3D). RNAs > 200 nt were not detected (S2A–S2D Fig). In no case was intact ribosomal RNA detected, indicating that detectable levels of cellular content were not contaminating the samples.

**Fig 4:**
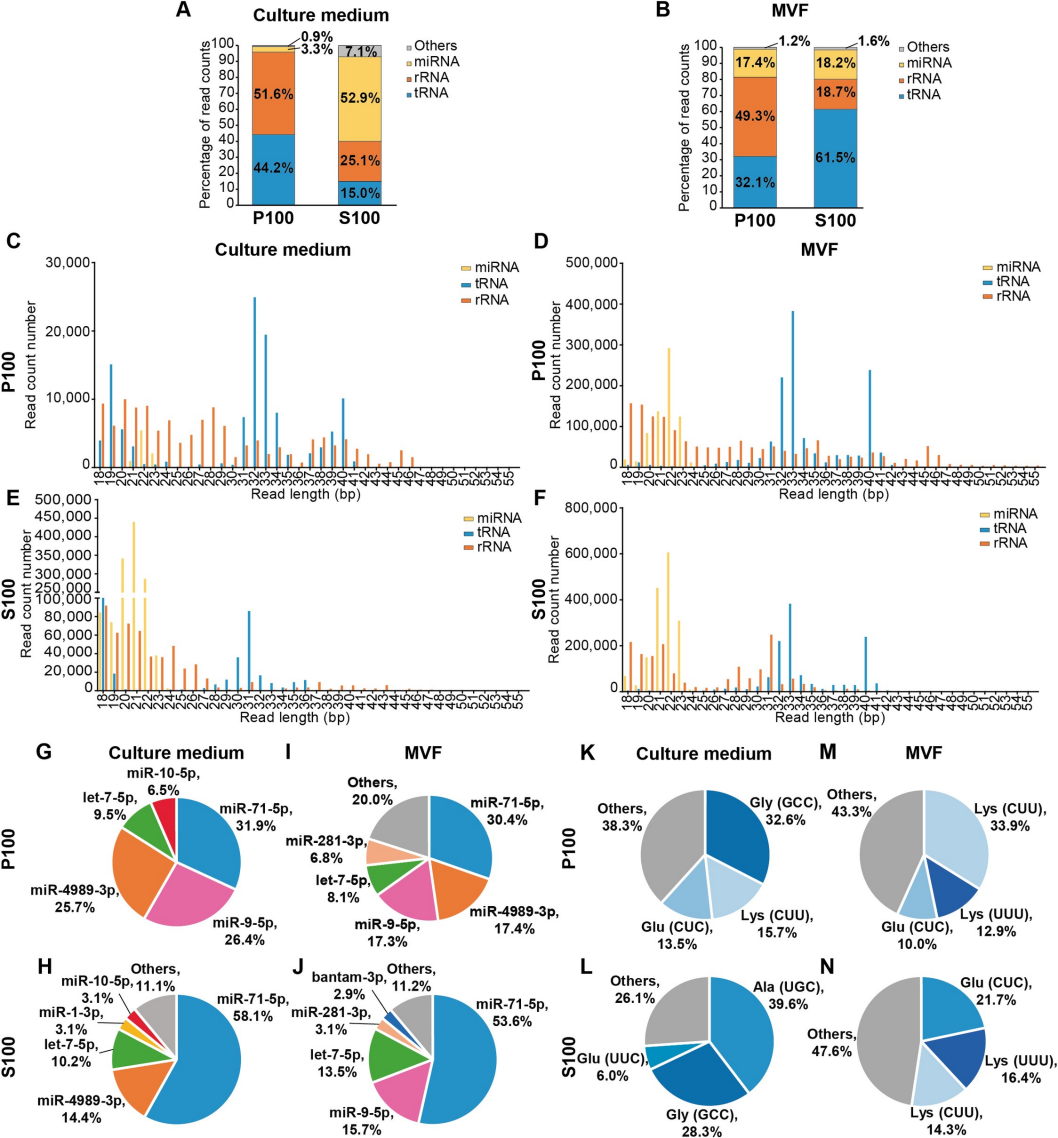
Ex-RNA profiling in P100 and S100 fractions from active metacestode cultures. RNA biotypes identified in culture medium (A) and metacestodes vesicular fluid (MVF) (B). Size distribution of miRNAs, tRNA-derived sequences and rRNA-derived sequences detected in culture medium (C, E) and MVF (D, F). Most abundantly detected miRNAs (G-J) and tRNA-derived sequences (K-N) from *E*. *multilocularis*.

However, ex-RNAs were identified by sRNA-seq in P100 and S100 fractions from culture medium and MVF (Table 1). This difference with respect to the results described above may be due to differences in the sensitivity of the techniques used. Most of the reads from each library mapped unambiguosly to the *E*. *multilocularis* genome (50.1% - 93.6%) and to a lesser extent clearly mapped to the *M*. *musculus* genome (0.1% - 8.4%). The size distribution of *E*. *multilocularis* mapping reads showed that P100 samples displayed three peaks: 19 bp, 32 bp and 40 bp for culture medium and 22 bp, 33 bp and 40 bp for MVF (S2E and S2F Fig). In S100 samples, peaks at 21 bp in culture medium and 22 bp and 33 bp in MVF were observed (S2G and S2H Fig). The 60-nt RNAs detected by high resolution electrophoresis in MVF (Fig 3C and 3D) were not present in the corresponding libraries (S2F and S2H Fig). Mapping reads corresponded mainly to three RNA biotypes: miRNAs, tRNA-derived sequences and rRNA-derived sequences (Fig 4A and 4B). In culture medium, sequences derived from tRNAs (44.2%) and rRNAs (51.6%) predominated in P100, while miRNA-mapping reads (52.9%) were the most abundant in S100. In MVF, sequences derived from rRNA (49.3%) or tRNAs (61.5%) were the most abundant in P100 and S100, respectively.

**Table 1:** General results of small RNA sequencing of ex-RNAs present in culture medium and metacestode vesicular fluid of active culture from *E*. *multilocularis*.

	Culture medium		Metacestode vesicular fluid	
	P100	S100	P100	S100
**Raw reads**	14,556,134	16,006,027	16,722,600	19,188,817
**Pre-processed reads**^**a**^	511,911	2,845,901	4,561,023	9,478,984
**Mapped unambiguously to the *E*. *multilocularis* genome**	256,506 (50.1%)	2,389,415 (84.0%)	3,907,687 (85.7%)	8,876,768 (93.6%)
**Mapped unambiguously to the *Mus musculus* genome**	7,483 (1.5%)	238,629 (8.4%)	4,545 (0.1%)	6,944 (0.1%)
**Mapped ambiguously to both genomes**	10,255 (2.0%)	9,254 (0.3%)	23,720 (0.5%)	28,627 (0.3%)
***E*. *multilocularis* miRNAs**^**b**^	12,399	1,274,335	719,761	1,670,829
**Vertebrate miRNAs**^**b**^	0	28,299	0	4,461
***E*. *multilocularis* tRNAs**	113,491	357,309	1,254,251	5,460,039
**Vertebrate tRNAs**	5,760	81,298	3,587	0
***E*. *multilocularis* rRNAs**	132,420	599,490	1,928,249	1,661,353
**Vertebrate rRNAs**	0	0	0	0

^a^ Pre-processed reads includes those with: no adaptors, PHRED ≥ 20, length ≥ 18 bp, ≥350 counts.

^b^ miRDeep prediction.

According to the criteria used in this work for RNA-seq data analysis, miRNAs from *E*. *multilocularis* were detected in all the extracellular compartments (Fig 4A and 4B). As expected, size distribution of miRNAs showed a peak at 21–22 bp in all samples (Fig 4C–4F). The S100 fraction of culture medium showed a 16-fold enrichment in *E*. *multilocularis* miRNAs with respect to the P100 fraction, while the P100 and S100 fractions of MVF showed almost the same proportion of miRNAs. Five and 19 miRNAs were detected in P100 and S100 from culture medium, respectively, while 22 and 20 miRNAs were detected in P100 and S100 from MVF, respectively (S2 and S3 Tables). miR-71-5p was the most abundantly detected in all samples followed by miR-9-5p and miR-4989-3p in the P100 fraction from both samples (Fig 4G and 4I); miR-4989-3p and let-7-5p in S100 from culture medium (Fig 4H) and miR-9-5p and let-7-5p in S100 from MVF (Fig 4J).

To assess the enrichment of miRNAs secreted to the extra-parasite compartment, the abundance of miRNAs present in the S100 fraction from culture medium was compared to expression levels in metacestode tissue (S3 Fig). Eight miRNAs showed enrichment in the extra-parasite milieu and three of them (miR-1-3p, miR-125-5p and miR-96-5p) had identical seed regions to their mouse homologues (S3B Fig).

**Fig 5:**
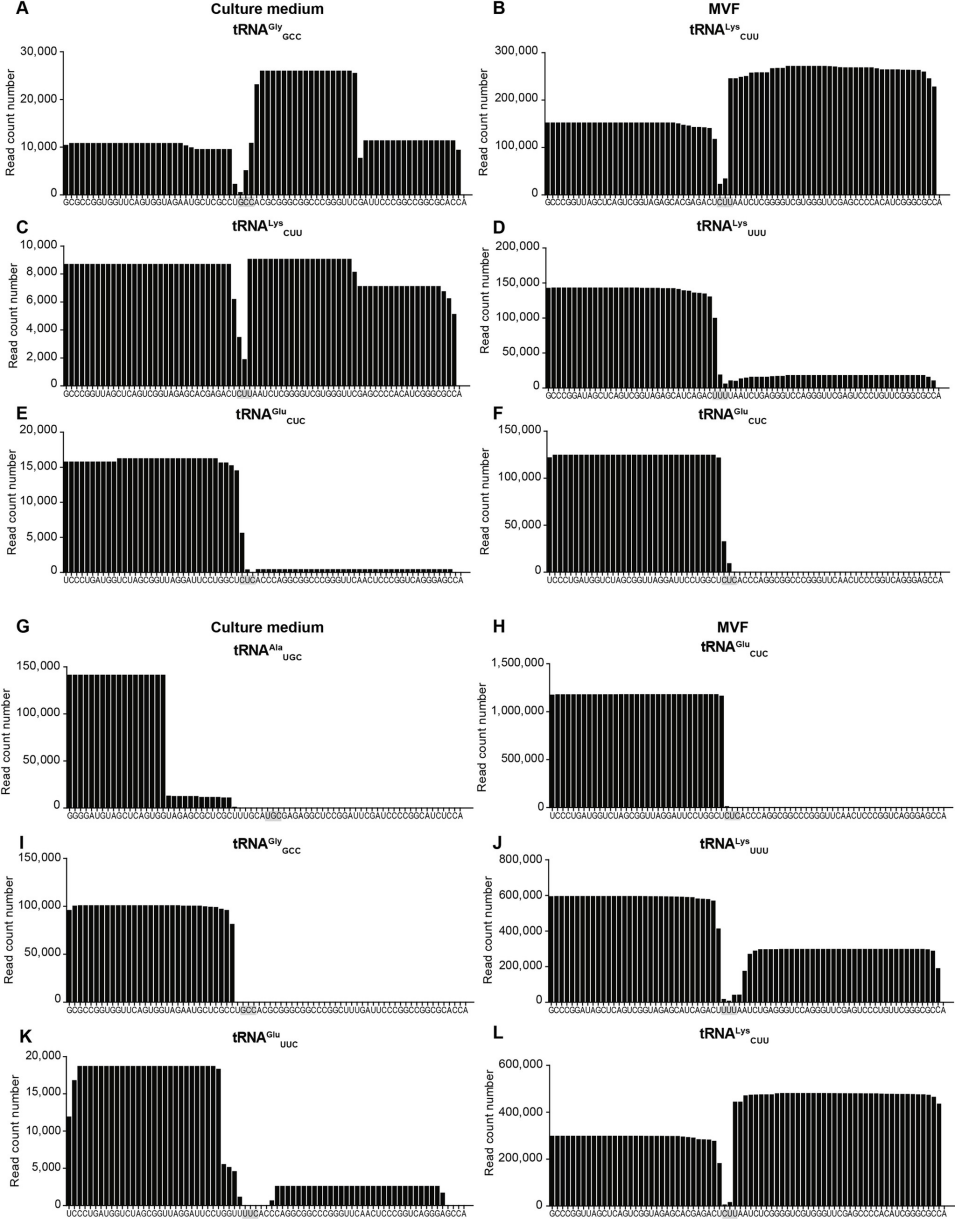
Length distribution of reads mapping to mature tRNAs of *E*. *multilocularis*. tRNA-derived sequences detected in the P100 (A-F) and S100 (G-L) fractions of culture medium and metacestode vesicular fluid (MVF) of active cultures. The sequence of each mature tRNA is shown.

Moreover, sequences derived from tRNAs of *E*. *multilocularis* displayed distinctive size distributions compatible with tRNA-derived fragments (~ 18–19 bp) and tRNA-halves (~ 32–33 bp) [49] (Fig 4C–4F). In each extracellular compartment, ≥ 50% of these tRNA-derived sequences mapped to two or three genes: in culture medium tRNA^Gly^_GCC_, tRNA^Lys^_CUU_ and tRNA^Glu^_CUC_ in P100 (Fig 4K) and tRNA^Ala^_UGC_ and tRNA^Gly^_GCC_ in S100 (Fig 4L); in MVF tRNA^Lys^_CUU_, tRNA^Lys^_UUU_ and tRNA^Glu^_CUC_ in P100 (Fig 4M) and tRNA^Glu^_CUC_, tRNA^Lys^_UUU_ and tRNA^Lys^_CUU_ in S100 (Fig 4N). The alignment of reads to each individual locus showed a mapping pattern compatible with sRNA formation (i.e., ≥ 80% of reads mapped to a specific site) for tRNA^Glu^_CUC_ in P100 (Fig 5E) and tRNA^Ala^_UGC_ and tRNA^Gly^_GCC_ in S100 (Fig 5G and 5I) from culture medium; and tRNA^Lys^_UUU_ and tRNA^Glu^_CUC_ in P100 (Fig 5D and 5F) and tRNA^Glu^_CUC_ in S100 (Fig 5H) in MVF. In all cases, the sRNAs originated from the 5´end of the tRNAs.

The reads mapping to *E*. *multilocularis* rRNA loci displayed no distinctive size pattern in P100 samples from culture medium or MVF, whereas a marked profile was observed for the S100 samples: a broad 18–26 bp distribution of sRNAs in culture medium (Fig 4C and 4E), and a bimodal distribution in MVF, with peaks at 18–21 bp and at 31 bp (Fig 4D and 4F). However, only in MVF the mapping pattern in three loci, with read lengths varying between 18–24 bp and 30–34 bp, showed strong evidence of sRNA biogenesis (S4 Table). This is due to the fact that sRNAs have a defined length and are generated from highly specific loci, features that are reflected in reproducible read profiles obtained by high-throughput sequencing which represent the originating processing mechanism [50,51].

Finally, an ex-RNA originating from the Signal Recognition Particle (SRP) RNA was observed in S100 from culture medium. SRP is a universally conserved ribonucleoprotein complex involved in targeting transmembrane and secretory proteins to the endoplasmic reticulum [52]. Here, we observed that an ex-RNA originating from the SRP RNA of *E*. *multilocularis* accounted for 6.9% of mapping reads and originated from the 3´end of the gene (S4A Fig).

With respect to reads mapping to the *M*. *musculus* genome, we only focused on those vertebrate sRNAs detected in the MVF. *Echinococcus multilocularis* metacestodes multiply asexually *in vitro* in co-culture with hepatocytes, which are removed when axenic parasites are needed [31]. However, vertebrate ex-RNAs non-specifically associated with the laminated layer may remain after axenization and detach during the incubation period for sample collection. In this way, in MVF most reads corresponded to tRNA-derived sequences (78.9%) in P100, while in S100 miRNAs predominated (68.2%) (S5A Fig). The only miRNAs detected were miR-122-5p and miR-21a-5p, with the former being the most abundant (S5D Fig and S5 Table). Other sRNA-like reads corresponded to the U2 gene in S100 samples (S5A Fig). Additionally, this sRNA showed 84% identity with the sequence of bovine miR-1246 (S5 Table).

### Tegument integrity conditions the release of nanoparticles to the extra-parasite milieu with the consequent detection of P100-associated RNA

**Fig 6:**
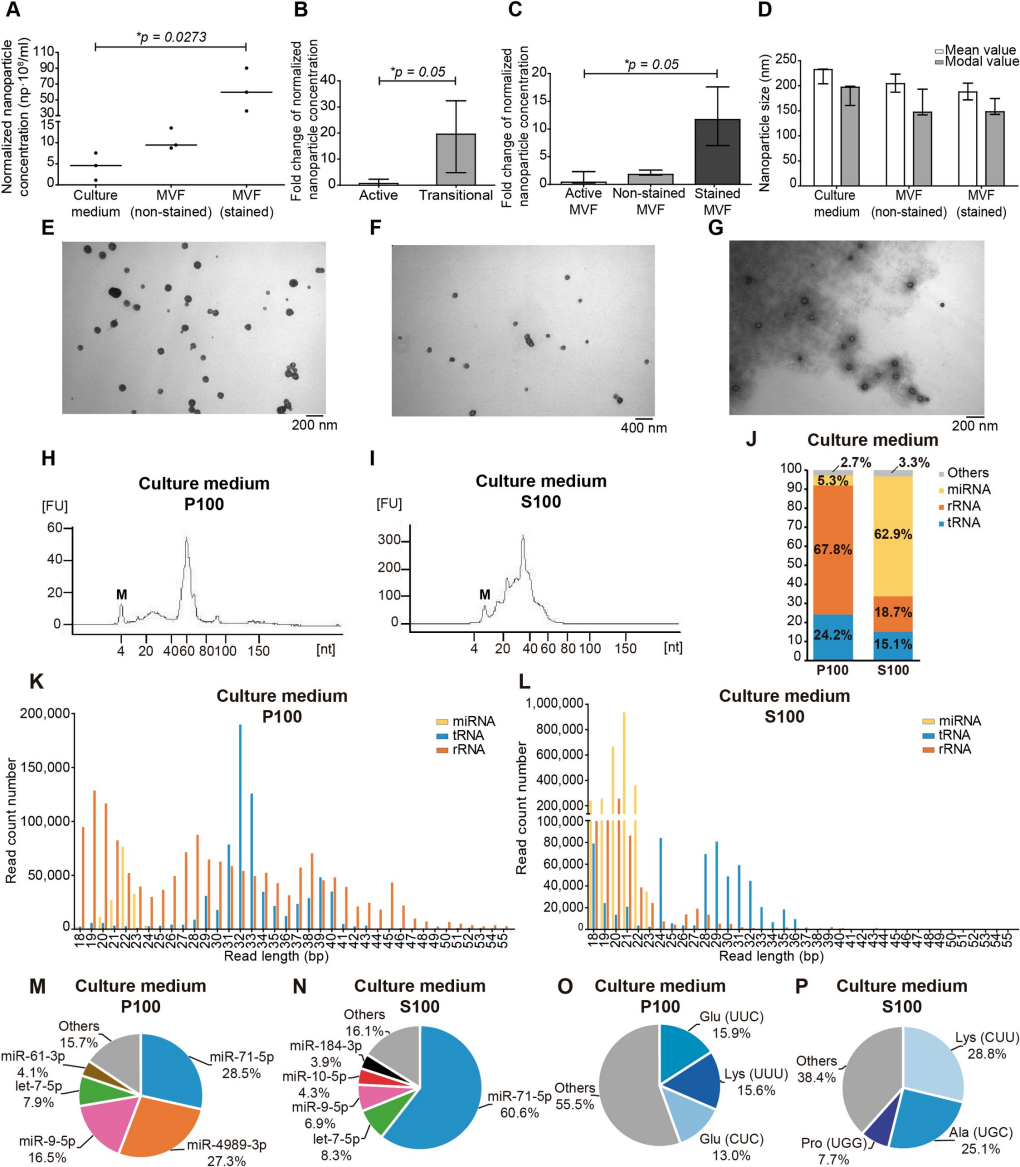
Characterisation of EV secretion and ex-RNAs secreted in transitional cultures. A) Nanoparticle tracking analysis of culture medium and metacestode vesicular fluid (MVF) from viable (non-stained) and senescent (stained) metacestodes. Normalized concentration of nanoparticles expressed in number of nanoparticles/ml. Each dot represents a replicate. Fold change of nanoparticle concentration detected in culture medium (B) and MVF (C) compared to active cultures. D) Mean and modal size of the detected nanoparticles. Bars indicate median size with range of the corresponding value. Transmission electron microscopy images of EV present in P100 from culture medium (E), non-stained (F) and stained (G) MVF. Analysis of the small RNA content (< 200 nt) present in the P100 (H) and S100 (I) fractions of culture medium. M: marker. FU: fluorescence units. J) RNA biotypes identified in the P100 and S100 fractions of culture medium. Size distribution of the three main RNA biotypes detected in the P100 (K) and S100 (L) fractions of culture medium. Most abundantly detected miRNAs (M, N) and tRNA-derived sequences (O, P) in culture medium.

In human infections, the *E*. *multilocularis* metacestode eventually develops as a heterogeneous multivesicular mass with regions of necrotic parasite tissue [16]. To study EV secretion in the progression towards this heterogeneous state, we analysed transitional cultures composed of active viable metacestodes and metacestodes with compromised tegument integrity (senescent parasites), as indicated by vital staining. In this way, we observed that the P100 fraction from medium of transitional cultures, with ~ 30% of senescent metacestodes, contained 4.58 x 10^8^ (1.11 x 10^8^–7.65 x 10^8^) nanoparticles/ml which represents an increase of ~ 20-fold with respect to P100 from the active cultures described above (p = 0.05) (Fig 6A and 6B). The detected nanoparticles had mean diameters of 232 nm (204–233), with no statistical difference with active culture medium values. Regarding MVF, metacestodes with disturbed tegument (stained MVF) secreted 5.98 x 10^9^ (3.60 x 10^9^–9.02 x 10^9^) nanoparticles/ml, i.e. a significant increase of ~12-fold nanoparticles with repect to MVF from active metacestodes (p = 0.05) (Fig 6A and 6C). Viable non-stained metacestodes from the transitional cultures showed no significant differences with respect to active culture, with 9.45 x 10^8^ (8.74 x 10^8^–13.49 x 10^8^) nanoparticles/ml secreted into the MVF (Fig 6A and 6B). Nanoparticle diameter did not significantly vary with respect to active cultures, with mean sizes of 204 nm (187–223) and 188 nm (172–188) for non-stained and stained samples, respectively (Fig 6D). EV presence could also be detected in all samples by transmission electron microscopy (Fig 6E–6G).

In contrast to active cultures, RNA could be readily detected by electrophoresis in all the extracellular compartments, including the P100 fraction from culture medium where the RNA size profile showed high similarity to that of MVF (Figs 6H, 6I and S6A–S6D, and S6I–S6N). As described in the previous section for active metacestode culture, the size distribution of sequenced reads did not exactly match the RNA size distribution. In this way, 60-nt RNAs were absent from the libraries (S6O–S6T Fig) as well as the 40-nt RNAs from the S100 library from culture medium (S6P Fig).

General sequencing results are shown in S6 Table. The main biotypes of ex-RNAs detected corresponded to miRNAs and sRNAs derived from tRNAs and rRNAs, with miRNAs constituting the most abundantly secreted RNA only in S100 from culture medium (Figs 6J and S7A and S7B). The size distribution of miRNAs and rRNA-derived sequences in culture medium resembled that from active culture samples, whereas tRNA-derived sequences showed some differences such as no peak at 19 bp in P100 and peaks at 24 bp and 28–29 bp in S100 (Fig 6K and 6L). With respect to MVF, non-stained samples resembled the distribution observed in active cultures for the three RNA biotypes, while stained samples did so in P100 and only for miRNAs in S100 since a peak at 18 bp in tRNA-derived sequences could be observed, and there was no peak at 31 bp in rRNA-derived sequences (S6E–S6H Fig).

In comparison to samples from active metacestode culture, P100 from transitional culture medium contained more miRNAs (16 vs 5) (S2 Table). Among the most abundantly detected miRNAs were those also detected in active cultures (Fig 6M and 6N). In S100 the number of miRNAs was similar (S2 Table). With respect to MVF, stained and non-stained samples yielded similar results compared to active cultures (S3 Table and S6C, S6E, S6G and S6I Fig), including the presence of vertebrate U2/miR-1246 and miRNAs only in the S100 samples, with miR-122-5p as the most frequently detected (S5 Table and S5B, S5C, S5E and S5F Fig).

As for sequences derived from tRNAs (S7 Table), sRNA generation was observed for 5´ end-tRNA^Glu^ RNAs, from one or two isoacceptors, in P100 samples (Figs 6O and S7D and S7H). In S100 the situation was more diversified, with the detection of 5´end-tRNA^Ala^_(UGC)_ in culture medium and stained MVF, and 5´end-tRNA^Pro^_(UGG)_ and 3´end-tRNA^Lys^_(CUU)_ in culture medium (Figs 6P and S7F and S7J).

Regarding the reads mapping to *E*. *multilocularis* rRNA, there was no observable distinctive size pattern in P100 samples, while in S100 there was a marked profile with sRNAs of 18–21 bp (Figs 6K–6L and S6E–S6H). Performing the analysis described in the previous section, we observed the presence of sRNAs generating from large and small subunit ribosomal RNA loci, in culture medium and MVF, respectively (S4 Table). In culture medium the source locus is the same as in the MVF from active cultures (URS000042B970_6211); however, the sRNA is shorter (20 vs 31 nt). In non-stained and stained MVF, the source locus (URS0000C65767_6211) and the sRNA generated are the same as in active cultures.

### *Echinococcus spp*. ex-RNAs can be detected in culture medium by RT-qPCR but not in plasma/sera from patients

**Fig 7:**
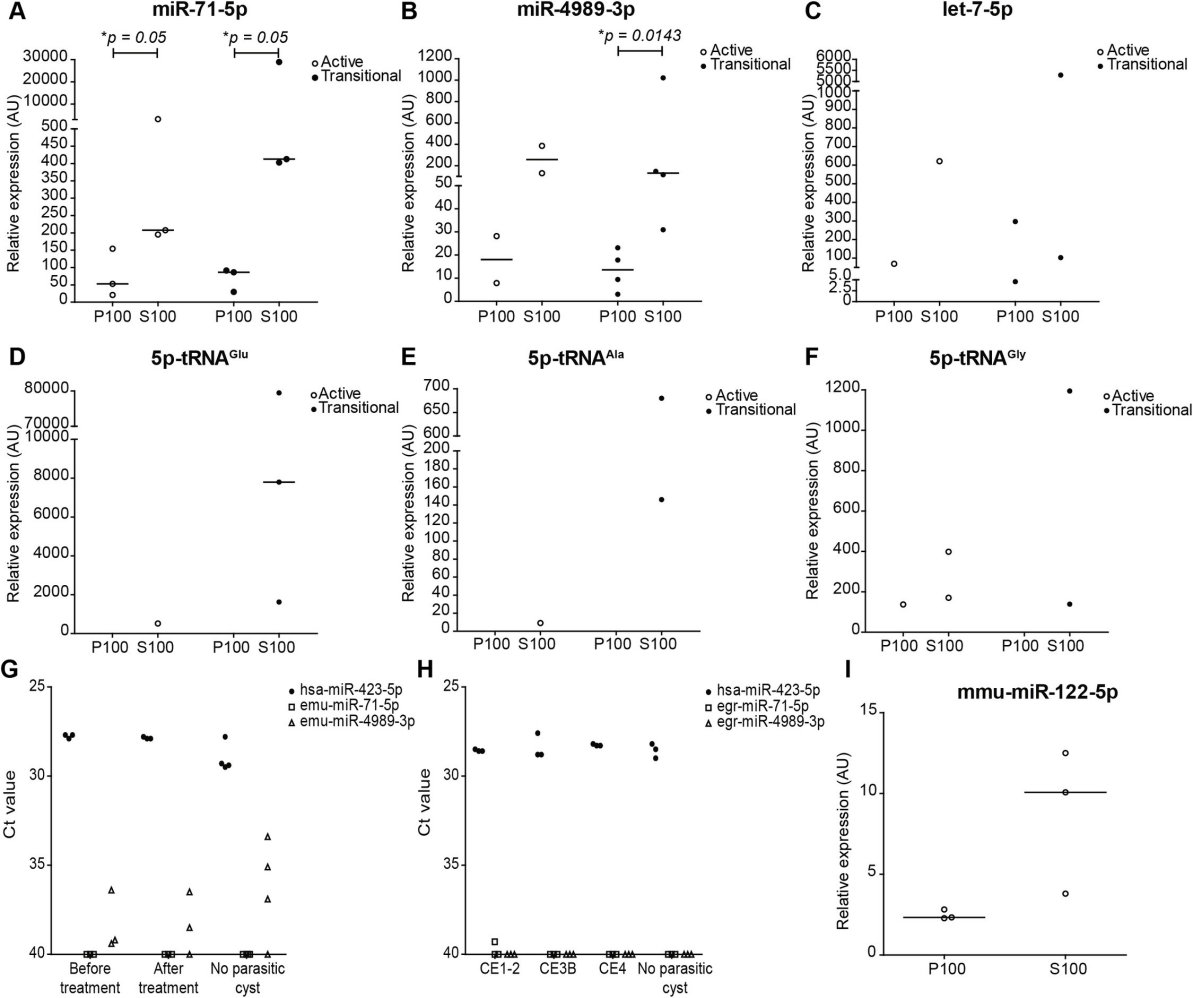
RT-qPCR detection of selected ex-RNAs *in vitro* and *in vivo*. A-F) Relative expression from selected miRNAs in culture medium from active and transitional cultures of *E*. *multilocularis* metacestodes is depicted in arbitrary units (AU). Values were normalised to input culture medium. Number of biological replicates tested for microRNAs: N Act = 3, N Trans = 4, and for tRNAs: N Act = 2, N Trans = 3. Assessment of detection of *Echinococcus* spp. microRNAs in serum/plasma samples from alveolar (G) and cystic (H) echinococcosis patients. Raw Ct values are shown. Ct = 40 means no amplification. I) Expression levels of vertebrate miR-122-5p in metacestode vesicular fluid from active cultures. Expression is based on RT-qPCR analysis of P100 and S100 fractions; values were normalized to input vesicular fluid. N = 3. Lines indicate the median values.

In order to determine if those ex-RNAs that were most abundantly secreted to the extra-parasite milieu (i.e., P100 and S100 from culture media) could be detected by RT-qPCR both *in vitro* and *in vivo*, we assessed culture medium samples as well as sera and plasma from patients with AE or CE. For this, we selected miR-71-5p, miR-4989-3p, let-7-5p and 5´-derived sRNAs of tRNA^Glu^, tRNA^Ala^ and tRNA^Gly^ for assessment in culture medium. As a result, miR-71-5p and miR-4989-3p were detected in most biological replicates (Fig 7A and 7B). Notably, tRNA-derived sRNAs were almost exclusively detected in S100 samples (Fig 7D–7F). Since miR-71-5p and miR-4989-3p are not encoded in mammalian genomes, we assessed patient samples. For miR-71-5p no amplification was observed in AE samples and only non-specific signal was detected for miR-4989-3p in all groups (Fig 7G). In CE samples, only one patient showed a very low amplification signal (Ct = 39.3) for miR-71-5p and no signal was observed for miR-4989-3p (Fig 7H). In all cases, the control endogenous miRNA (hsa-miR-423-5p) was successfully detected.

Finally, the presence of host miR-122-5p could be detected in MVF (Fig 7I).

## Discussion

In this work, we have characterised the profile of ex-RNA secreted *in vitro* by *E*. *multilocularis* metacestodes. Since this larval stage is a fluid-filled vesicle, the germinal layer has an apical surface that secretes molecules towards the extra-parasite milieu (culture medium) and a basal surface whose secretions accumulate in the intra-parasite milieu (MVF). One such type of molecules are nucleic acids which have been described in other organisms to be secreted in soluble ribonucleoprotein complexes or associated to EV or lipoproteins. Previously, we have determined by transmission electron microscopy that EV could be hardly detected in the culture medium of *E*. *multilocularis* metacestodes in contrast to media conditioned by metacestodes from *T*. *crassiceps* and *M*. *corti* [24]. In that work, we also observed that EV from diverse sizes were located in the interface between the germinal and laminated layers. Here, we used the more sensitive technique NTA to quantify the number of nanoparticles released to both the culture medium and the MVF. This analysis was conducted in active cultures composed of metacestodes with no tegument impairment and in transitional cultures with first signs of senescence.

In this way, we observed in active cultures that EV are mainy secreted towards the intra-parasite milieu since a significant higher number of nanoparticles were present in the MVF. However, in transitional cultures there was an increase of more than 10-fold in the number of nanoparticles detected in the extra-parasite milieu and the MVF of senescent metacestodes. As the tegument is responsible for synthesizing the laminated layer [17], we hypothesize that the ultrastructure of the latter is compromised by tegument impairment, allowing the passage of the nanoparticles mainly retained in the proximity of the germinal layer [24]. This is of particular interest to understand the parasite progression in human natural infections where the metacestode harbors necrotic regions [16]. Due to the fact that *Echinococcus* spp. EV can alter the cellular and functional phenotypes of immune cells *in vitro* [27,28], *E*. *multilocularis* EV release *in vivo* may represent a strategy to divert the immune system while allowing parasite survival through stem cell metastasis [53]. In addition, given that tegument impairment may be associated with cellular damage, it would be interesting to study if the extra-parasite secretion of larger EV populations, that were not the focus of the present work, also increases upon parasite transition to senescence.

In line with these observations, ex-RNAs in active cultures were enriched in the S100 (EV-depleted) fraction of culture medium in comparison to the P100 (EV-enriched) fraction, while in transitional cultures ex-RNAs could be abundantly detected in the P100 fraction. This implies that in undisrupted metacestodes ex-RNAs may be secreted in soluble ribonucleoprotein complexes or lipoproteins. It is known that *Echinococcus* spp. metacestodes release proteins to the extra-parasite medium *in vivo* due to the presence of host circulating antibodies generated against parasite antigens [14]; however, data on their capacity to bind RNA is still lacking. It is worth mentioning that future work involving RNAse protection assays will help to better understand the form in which S100 ex-RNAs are secreted. Interestingly, it was recently shown that *Trichinella spiralis* muscle stage larvae *in vitro* secrete unprotected sRNAs suggesting the existence of an alternative RNA secretion mechanism than those described to date [48] that may also apply to other helminths.

In this work, we demonstrated that *E*. *multilocularis* metacestodes mainly secrete sRNAs (< 200 nt) in agreement with previous reports on ex-RNAs present in EV-enriched and EV-depleted samples from medium conditioned by helminth parasites [19,21,24,54]. With respect to those sRNAs that could be detected by capillary electrophoresis but were not present in the libraries, we can infer that they had structural and/or chemical modifications that imposed constraints to the adapter ligation protocol used in this work that takes advantage of the 5´ PO_4_^-^ and 3´OH^-^ present in miRNAs and tRNAs [55], since no size selection was performed in library construction.

The ex-RNA profile from the S100 samples of culture media showed a predominant proportion of miRNAs in contrast to observations made on ex-RNAs from mammalian cell lines [33,56,57]. This high content of miRNAs was also observed in S100 from culture media conditioned by L4 larvae of *Haemonchus contortus* but not in S100 nor P100 from adults [22]. Another report on secretions of adults of *H*. *polygyrus* shows an inverse proportion, with 75% reads mapping to miRNAs in P100 and only 10% in S100 [21]. Although more information is needed to reach conclusions for each parasite class, current data suggests a varying ex-RNA scenario depending on life cycle stage and parasite species.

Previously, we reported that miR-71-5p has a moderate expression level in *E*. *multilocularis* metacestodes from experimental infections accounting for 8.7% (± 0.27) of total miRNA expression [40]. Here, it was consistently detected in P100 and S100 samples from culture media and MVF by sRNA-seq, and confirmed by RT-qPCR to be enriched in S100 from both active and transitional cultures. In addition, miR-71-5p was found to be enriched in the S100 fraction of active culture medium compared to expression levels in metacestodes developed *in vivo* [40]. Even though the extracellular miRNA levels were obtained from *in vitro* generated metacestodes, these results suggest that not only the most abundant miRNAs expressed in parasite tissues are secreted. miR-71-5p and miR-4989-3p have been detected in cestodes EV [24,30,58] and according to our results, they are the most abundant and divergent ex-RNAs that could be used as biomarkers to detect the presence of active metacestodes. However, our preliminary tests in serum and plasma samples from patients with AE and CE with hepatic location did not yield encouraging results, since we could not confidently detect these miRNAs. Similarly, it was reported that miRNAs from the intestinal parasite *H*. *polygyrus* could not be detected in sera from infected mice while miRNAs from the circulating filarial nematode *Litomosoides sigmodontis* could [21]. In addition, no miRNAs from *H*. *contortus* could be detected in serum from infected sheep [59] although they could be detected in tissue at the infection site [22]. Altogether, these data suggest that detection in serum or plasma depends on parasite species and its location within the host. Other authors have reported the detection of circulating *E*. *multilocularis* miRNAs in sera from experimentally infected mice [60]; however, the infection conditions (route of inoculation, dose, parasite stage, site of parasite development) highly differ between natural and experimental infections to reach a firm conclusion.

Several parasite miRNAs enriched in the extra-parasite S100 fraction share the seed region with their homologous host miRNAs, suggesting that the parasite could mimic those miRNAs to regulate the same pathways. Particularly, miR-96-5p was reported to regulate cholesterol uptake and biosynthesis [61,62]. Given that cestode parasites lack the machinery to synthesize fatty acids and cholesterol *de novo* [63], it would be interesting to study the role of emu-miR-96-5p in modulating cholesterol availability for parasite use. Another interesting miRNA is miR-125-5p, whose expression was described to be increased in fibrotic liver and to negatively regulate the factor inhibiting hypoxia-inducible factor 1 (HIF1) [64]. HIF1 is a master transcription factor that regulates genes involved in angiogenesis, among others. Since the metacestode of *E*. *multilocularis* infiltrates and destroys the host liver parenchyma, liver fibrosis is a common pathological feature of AE which is also associated with abundant vascular structures proximal to the parasite [65]. In this way, secretion of miR-125-5p could be a strategy to increase nutrient supply to the parasite through neovascularization.

Interestingly, we confirmed the presence of host miRNAs in the intra-parasite milieu, implying their transport through the tegument towards the MVF. In particular, the most abundant miRNA was miR-122-5p. This miRNA is not encoded in invertebrate genomes, it has liver-enriched expression and it is known to be secreted and present in bovine serum [66]. The asexual multiplication of *E*. *multilocularis* metacestodes *in vitro* is carried out in co-culture with rat hepatocytes in the presence of fetal bovine serum [31]. Due to the fact that the sequence of miR-122-5p is identical between rat and cow we cannot determine the origin of this miRNA, but to the best of our knowledge, this is the first report on the internalization of host RNAs in an helminth parasite. Future experiments will determine how this miRNA is transported through the parasite tegument and if it plays a role on parasite establishment, development and/or survival.

With respect to other ex-sRNAs, tRNA-derived sequences were found. These sequences mapped mainly to the 5´end of certain tRNAs, such as tRNA^Glu^, tRNA^Gly^ and tRNA^Ala^. We further confirmed by RT-qPCR that these sRNAs were detected in S100 from culture medium, in agreement with findings in human cell lines, where tRNA-derived sRNAs were predominantly detected in EV-depleted fractions and mostly corresponded to tRNA^Glu^ and tRNA^Gly^ [33,56]. In parasitic nematodes no predominance of tRNA-derived sequences in secretion products (EV-enriched or EV-depleted) was observed [21,22], whereas in platyhelminths, the presence of tRNA-derived sequences in both fractions was reported in culture medium of schistosomula from *Schistosoma mansoni* though their relative abundance in relation to other RNA biotypes was not assessed [23]. tRNA-derived sRNAs are produced when cells are under stress conditions and although the mechanisms underlying the mode of action of this class of sRNAs is still not completely understood, some of their described roles involve the regulation of gene expression in a miRNA-like manner, the displacement of translation initiation factors from target mRNAs and the control of transposable elements to maintain genome stability [10,67]. Our results point to a new player in the communication between parasite and host and also, between parasite cells due to the presence of these sRNAs in the MVF.

Here we have shown that *in vitro* and in the absence of host stimuli, a polarized secretion of sRNAs takes place in the non-vesicular fraction (S100) of *E*. *multilocularis* metacestodes, with miRNAs mainly secreted to the extra-parasite milieu and rRNA- and tRNA-derived sequences secreted to the intra-parasite milieu. Further studies are necessary to determine if *E*. *multilocularis* ex-RNAs play a role in targeting neighbouring host cells and if they can efficiently reach the host bloodstream, for instance, in early metacestodes with a still non consolidated laminated layer.

We believe that a comprehensive study of the secretion mechanisms throughout the life cycle of these parasites will help to improve current diagnostic tools by targeting those molecules with higher chances to be detected in host body fluids according to parasite state of development.

## Supporting information

S1 Fig Phenol red evaluation as indicator of parasite integrity. A) Classification of Active and Transitional metacestodes according to phenol red staining (final concentration 0.04 mM) and eosin staining (final concentration 0.02%). B) Collapsed metacestodes with loss of turgency were excluded from cultures. White arrows show collapsed metacestodes. Scale bars indicate 1 cm.
(TIF)

S2 Fig RNA and read size distributions in active cultures. Analysis of the long RNA (> 200 nt) content present in the P100 and S100 fractions of culture medium (A, C) and metacestode vesicular fluid (B, D) of active *E*. *multilocularis* metacestodes. M: marker. FU: fluorescence units. Size distribution of reads mapping unambiguously to the *E*. *multilocularis* genome detected in the P100 and S100 fractions of culture medium (E, G) and metacestodes vesicular fluid (F, H) of metacestodes.
(TIF)

S3 Fig Enrichment analysis of miRNAs present in fraction S100 of active culture medium. (A) Linear regression of miRNA abundance in fraction S100 of active culture medium and in metacestode tissue. Each dot represents a miRNA. Red dots indicate miRNAs enriched in S100 fraction of active culture medium; hollow dots indicate miRNAs enriched in metacestode tissue; black dots indicate miRNAs with equal abundance. (B) miRNAs enriched in the S100 fraction of active culture medium. The sequence, *M*. *musculus* homologous miRNAs and level of enrichment are shown.
(TIF)

S4 Fig sRNA derived from the *E*. *multilocularis* Signal Recognition Particle (SRP) RNA. A) Length distribution of reads detected in culture medium of active metacestodes. Sequences corresponding to reads mapping to one specific region (±1 nt) and account for ≥ 50% of total read counts for this gene are shown. Only the 3´-end of the gene is displayed. B) RT-qPCR detection of SRP-derived sequence in P100 and S100 from active and transitional cultures. N = 3 each. Lines indicate the median values.
(TIF)

S5 Fig Host ex-RNA biotypes found in the vesicular fluid of *E*. *multilocularis* metacestodes. Analysis of the P100 and S100 fractions of metacestode vesicular fluid of *E*. *multilocularis* active (A) and transitional (B, C) cultures. Host microRNAs detected in the S100 fraction from metacestode vesicular fluid of active (D) and transitional (E, F) cultures.
(TIF)

S6 Fig RNA and read size distributions in transitional cultures. Analysis of the small RNA content (< 200 nt) present in the P100 and S100 fractions of metacestodes vesicular fluid (MVF) from viable (non-stained) (A, B) and senescent (stained) (C, D) metacestodes. Size distribution of the three main RNA biotypes detected in MVF from viable (E, F) and senescent (G, H) metacestodes. Analysis of the large RNA content (> 200 nt) present in the P100 and S100 fractions of culture medium (I, J) and MVF (K-N). M: marker. FU: fluorescence units. General size distribution of reads mapping unambiguously to the *E*. *multilocularis* genome detected in culture medium (O, P) and MVF (Q-T).
(TIF)

S7 Fig Ex-RNA profiling in metacestode vesicular fluid (MVF) of metacestodes from transitional cultures. RNA biotypes identified in the P100 and S100 fractions of MVF from viable (non-stained) (A) and senescent (stained) (B) metacestodes. Most abundantly detected miRNAs (C, E, G, I) and tRNA-derived sequences (D, F, H, J) in non-stained and stained MVF.
(TIF)

S1 Table Primers used for reverse transcription (RT) and real-time PCR. (DOCX)

S2 Table *Echinococcus multilocularis* microRNAs detected in the EV-enriched (P100) and EV-depleted (S100) fractions of culture medium from active and transitional metacestode cultures. (XLSX)

S3 Table *Echinococcus multilocularis* microRNAs detected in the EV-enriched (P100) and EV-depleted (S100) fractions of metacestode vesicular fluid (MVF) from active and transitional metacestode cultures. (XLSX)

S4 Table *Echinococcus multilocularis* rRNA-derived small RNAs detected in S100 fractions. (DOCX)

S5 Table Vertebrate (*Mus musculus*) microRNAs detected in the EV-depleted (S100) fraction of metacestode vesicular fluid (MVF) from active and transitional metacestode cultures. (XLSX)

S6 Table General results of small RNA sequencing of ex-RNAs present in culture medium and metacestode vesicular fluid (MVF) of *E*. *multilocularis* transitional cultures. (DOCX)

S7 Table *Echinococcus multilocularis* tRNA-derived sequences. (XLSX)

## References

[1] Stroun M, Anker P, Maurice P, Gahan PB. Circulating Nucleic Acids in Higher Organisms. Int Rev Cytol. 1977;51(C):1–48. 10.1016/s0074-7696(08)60225-9 338535

[2] Stroun M, Anker P, Belianski M, Henri J, Lederrey C, Ojha M, et al Presence of RNA in the Nucleoprotein Complex Spontaneously Released by Human Lymphocytes and Frog Auricles in Culture. Cancer Res. 1978;38(10):3546–54. 688240

[3] Wieczorek AJ, Rhyner C, Block LH. Isolation and characterization of an RNA-proteolipid complex associated with the malignant state in humans. Proc Natl Acad Sci U S A. 1985;82(10):3455–9. 10.1073/pnas.82.10.3455 2582412 PMC397794

[4] Wang K, Zhang S, Weber J, Baxter D, Galas DJ. Export of microRNAs and microRNA-protective protein by mammalian cells. Nucleic Acids Res. 2010;38(20):7248–59. 10.1093/nar/gkq601 20615901 PMC2978372

[5] Arroyo JD, Chevillet JR, Kroh EM, Ruf IK, Pritchard CC, Gibson DF, et al Argonaute2 complexes carry a population of circulating microRNAs independent of vesicles in human plasma. Proc Natl Acad Sci U S A. 2011;108(12):5003–8. 10.1073/pnas.1019055108 21383194 PMC3064324

[6] Turchinovich A, Weiz L, Langheinz A, Burwinkel B. Characterization of extracellular circulating microRNA. Nucleic Acids Res. 2011;39(16):7223–33. 10.1093/nar/gkr254 21609964 PMC3167594

[7] Vickers KC, Palmisano BT, Shoucri BM, Shamburek RD, Remaley AT. MicroRNAs are transported in plasma and delivered to recipient cells by high-density lipoproteins. Nat Cell Biol. 2011;13(4):423–33. 10.1038/ncb2210 21423178 PMC3074610

[8] Valadi H, Ekström K, Bossios A, Sjöstrand M, Lee JJ, Lötvall JO. Exosome-mediated transfer of mRNAs and microRNAs is a novel mechanism of genetic exchange between cells. Nat Cell Biol. 2007;9(6):654–9. 10.1038/ncb1596 17486113

[9] Tosar JP, Gámbaro F, Darré L, Pantano S, Westhof E, Cayota A. Dimerization confers increased stability to nucleases in 5’ halves from glycine and glutamic acid tRNAs. Nucleic Acids Res. 2018;46(17):9081–93. 10.1093/nar/gky495 29893896 PMC6158491

[10] Dou S, Wang Y, Lu J. Metazoan tsRNAs: Biogenesis, evolution and regulatory functions. Non-coding RNA. 2019;5(1):18 10.3390/ncrna5010018 30781726 PMC6468576

[11] Bartel DP. Metazoan MicroRNAs. Cell. 2018;173(1):20–51. 10.1016/j.cell.2018.03.006 29570994 PMC6091663

[12] Hoy AM, Lundie RJ, Ivens A, Quintana JF, Nausch N, Forster T, et al Parasite-derived microRNAs in host serum as novel biomarkers of helminth infection. PLoS Negl Trop Dis. 2014;8(2):e2701 10.1371/journal.pntd.0002701 24587461 PMC3930507

[13] Quintana JF, Makepeace BL, Babayan S a, Ivens A, Pfarr KM, Blaxter M, et al Extracellular *Onchocerca*-derived small RNAs in host nodules and blood. Parasites {&} vectors. 2015;8(1):58 10.1186/s13071-015-0656-1 25623184 PMC4316651

[14] Gottstein B, Wang J, Blagosklonov O, Grenouillet F, Millon L, Vuitton DA, et al *Echinococcus* metacestode: in search of viability markers. Parasite. 2014;21:63 10.1051/parasite/2014063 25429386 PMC4245873

[15] Wen H, Vuitton L, Tuxun T, Li J, Vuitton DA, Zhang W, et al Echinococcosis: Advances in the 21st century. Clinical Microbiology Reviews. 2019;32(2):e00075–18. 10.1128/CMR.00075-18 30760475 PMC6431127

[16] Thompson RCA. Biology and Systematics of *Echinococcus*. Vol. 95, Advances in parasitology. 2017;p. 65–109. 10.1016/bs.apar.2016.07.001 28131366

[17] Díaz A, Casaravilla C, Irigoín F, Lin G, Previato J, Ferreira F. Understanding the laminated layer of larval *Echinococcus* I: structure. Trends Parasitol. 2011;27(5):204–13. 10.1016/j.pt.2010.12.012 21257348

[18] Brunetti E, Kern P, Vuitton DA, Writing Panel for the WHO-IWGE. Expert consensus for the diagnosis and treatment of cystic and alveolar echinococcosis in humans. Acta Trop. 2010;114(1):1–16. 10.1016/j.actatropica.2009.11.001 19931502

[19] Bernal D, Trelis M, Montaner S, Cantalapiedra F, Galiano A, Hackenberg M, et al Surface analysis of *Dicrocoelium dendriticum*. The molecular characterization of exosomes reveals the presence of miRNAs. J Proteomics. 2014;105:232–41. 10.1016/j.jprot.2014.02.012 24561797

[20] Sotillo J, Robinson M, Kimber M, Cucher M, Ancarola ME, Nejsum P, et al The protein and microRNA cargo of extracellular vesicles from parasitic helminths—current status and research priorities. Int J Parasitol. 2020;50(9):635–645. 10.1016/j.ijpara.2020.04.010 32652128

[21] Buck AH, Coakley G, Simbari F, McSorley HJ, Quintana JF, Le Bihan T, et al Exosomes secreted by nematode parasites transfer small RNAs to mammalian cells and modulate innate immunity. Nat Commun. 2014;5:5488 10.1038/ncomms6488 25421927 PMC4263141

[22] Gu HY, Marks ND, Winter AD, Weir W, Tzelos T, McNeilly TN, et al Conservation of a microRNA cluster in parasitic nematodes and profiling of miRNAs in excretory-secretory products and microvesicles of *Haemonchus contortus*. PLoS Negl Trop Dis. 2017;11(11). 10.1371/journal.pntd.0006056 29145392 PMC5709059

[23] Nowacki FC, Swain MT, Klychnikov OI, Niazi U, Ivens A, Quintana JF, et al Protein and small non-coding RNA-enriched extracellular vesicles are released by the pathogenic blood fluke *Schistosoma mansoni*. J Extracell Vesicles. 2015;4:1–16. 10.3402/jev.v4.28665 26443722 PMC4595467

[24] Ancarola ME, Marcilla A, Herz M, Macchiaroli N, Pérez M, Asurmendi S, et al Cestode parasites release extracellular vesicles with microRNAs and immunodiagnostic proteins cargo. Int J Parasitol. 2017;47(10–11):675–86. 10.1016/j.ijpara.2017.05.003 28668323

[25] Siles-Lucas M, Sánchez-Ovejero C, González-Sánchez M, González E, Falcón-Pérez JM, Boufana B, et al Isolation and characterization of exosomes derived from fertile sheep hydatid cysts. Vet Parasitol. 2017;236:22–33. 10.1016/j.vetpar.2017.01.022 28288760

[26] Santos GB do., Monteiro KM, da Silva ED, Battistella ME, Ferreira HB, Zaha A. Excretory/secretory products in the *Echinococcus granulosus* metacestode: is the intermediate host complacent with infection caused by the larval form of the parasite? Int J Parasitol. 2016;46(13–14):843–56. 10.1016/j.ijpara.2016.07.009 27771257

[27] Zhou X, Wang W, Cui F, Shi C, Ma Y, Yu Y, et al Extracellular vesicles derived from *Echinococcus granulosus* hydatid cyst fluid from patients: isolation, characterization and evaluation of immunomodulatory functions on T cells. Int J Parasitol. 2019;49(13–14):1029–37. 10.1016/j.ijpara.2019.08.003 31734339

[28] Nicolao MC, Rodriguez Rodrigues C, Cumino AC. Extracellular vesicles from *Echinococcus granulosus* larval stage: Isolation, characterization and uptake by dendritic cells. PLoS Negl Trop Dis. 2019;13(1). 10.1371/journal.pntd.0007032 30615613 PMC6344059

[29] Zheng Y, Guo X, Su M, Guo A, Ding J, Yang J, et al Regulatory effects of *Echinococcus multilocularis* extracellular vesicles on RAW264.7 macrophages. Vet Parasitol. 2017;235:29–36. 10.1016/j.vetpar.2017.01.012 28215864

[30] Ding J, He G, Wu J, Yang J, Guo X, Yang X, et al miRNA-seq of *Echinococcus multilocularis* Extracellular Vesicles and Immunomodulatory Effects of miR-4989. Front Microbiol. 2019;10:1–9. 10.3389/fmicb.2019.00001 31849869 PMC6895134

[31] Spiliotis M, Brehm K. Axenic in vitro cultivation of *Echinococcus multilocularis* metacestode vesicles and the generation of primary cell cultures. Methods Mol Biol. 2009;470:245–62. 10.1007/978-1-59745-204-5_17 19089387

[32] Théry C, Amigorena S, Raposo G, Clayton A. Isolation and Characterization of Exosomes from Cell Culture Supernatants. Curr Protoc cell Biol. 2006;3:3.22.1–3.22.29. 10.1002/0471143030.cb0322s30 18228490

[33] Tosar JP, Gámbaro F, Sanguinetti J, Bonilla B, Witwer KW, Cayota A. Assessment of small RNA sorting into different extracellular fractions revealed by high-throughput sequencing of breast cell lines. Nucleic Acids Res. 2015;43(11):5601–16. 10.1093/nar/gkv432 25940616 PMC4477662

[34] Martin M. Cutadapt removes adapter sequences from high-throughput sequencing reads. EMBnet.journal. 2011;17(1):10.

[35] Bushnell B, Rood J, Singer E. BBMerge–Accurate paired shotgun read merging via overlap. PLoS One. 2017;12(10). 10.1371/journal.pone.0185056 29073143 PMC5657622

[36] Bolger AM, Lohse M, Usadel B. Trimmomatic: a flexible trimmer for Illumina sequence data. Bioinformatics. 2014;30(15):2114–20. 10.1093/bioinformatics/btu170 24695404 PMC4103590

[37] Friedländer MR, Mackowiak SD, Li N, Chen W, Rajewsky N. MiRDeep2 accurately identifies known and hundreds of novel microRNA genes in seven animal clades. Nucleic Acids Res. 2012;40(1):37–52. 10.1093/nar/gkr688 21911355 PMC3245920

[38] Macchiaroli N, Cucher M, Zarowiecki M, Maldonado L, Kamenetzky L, Rosenzvit MC. microRNA profiling in the zoonotic parasite *Echinococcus canadensis* using a high-throughput approach. Parasit Vectors. 2015;8(1):83 10.1186/s13071-015-0686-8 25656283 PMC4326209

[39] Cucher M, Prada L, Mourglia-Ettlin G, Dematteis S, Camicia F, Asurmendi S, et al Identification of *Echinococcus granulosus* microRNAs and their expression in different life cycle stages and parasite genotypes. Int J Parasitol. 2011;41(3):439–48. 10.1016/j.ijpara.2010.11.010 21219906

[40] Cucher M, Macchiaroli N, Kamenetzky L, Maldonado L, Brehm K, Rosenzvit MC. High-throughput characterization of *Echinococcus* spp. metacestode miRNomes. Int J Parasitol. 2015;45(4):253–67. 10.1016/j.ijpara.2014.12.003 25659494

[41] Friedländer MR, Chen W, Adamidi C, Maaskola J, Einspanier R, Knespel S, et al Discovering microRNAs from deep sequencing data using miRDeep. Nat Biotechnol. 2008;26(4):407–15. 10.1038/nbt1394 18392026

[42] Langmead B, Salzberg SL. Fast gapped-read alignment with Bowtie 2. Nat Methods. 2012;9(4):357–9. 10.1038/nmeth.1923 22388286 PMC3322381

[43] Camacho C, Coulouris G, Avagyan V, Ma N, Papadopoulos J, Bealer K, et al BLAST+: Architecture and applications. BMC Bioinformatics. 2009;10 10.1186/1471-2105-10-421 20003500 PMC2803857

[44] Lowe TM, Chan PP. tRNAscan-SE On-line: integrating search and context for analysis of transfer RNA genes. Nucleic Acids Res. 2016;44(W1):W54–7. 10.1093/nar/gkw413 27174935 PMC4987944

[45] Helbig M, Frosch P, Kern P, Frosch M. Serological differentiation between cystic and alveolar echinococcosis by use of recombinant larval antigens. J Clin Microbiol. 1993;31(12):3211–5. 10.1128/JCM.31.12.3211-3215.1993 8308113 PMC266377

[46] Ruijter JM, Ramakers C, Hoogaars WMH, Karlen Y, Bakker O, Hoff MJB Van Den, et al Amplification efficiency: linking baseline and bias in the analysis of quantitative PCR data. Nucleic Acids Res. 2009; 37(6):e45 10.1093/nar/gkp045 19237396 PMC2665230

[47] Pfaffl MW. A new mathematical model for relative quantification in real-time RT-PCR. Nucleic Acids Res. 2001; 29(9):45e–45. 10.1093/nar/29.9.e45 11328886 PMC55695

[48] Taylor PJ, Hagen J, Faruqu FN, Al-Jamal KT, Quigley B, Beeby M, et al *Trichinella spiralis* secretes abundant unencapsulated small RNAs with potential effects on host gene expression. Int J Parasitol. 2020;50(9):697–705. 10.1016/j.ijpara.2020.05.008 32622688 PMC7445429

[49] Lalaouna D, Carrier MC, Massé E. Every little piece counts: the many faces of tRNA transcripts. Transcription. 2015;6(4):74–7. 10.1080/21541264.2015.1093064 26595434 PMC4802806

[50] Langenberger D, Bermudez-Santana CI, Stadler PF, Hoffmann S. Identification and classification of small RNAS in transcriptome sequence data. In: Pacific Symposium on Biocomputing 2010, PSB 2010. 2010 p. 80–7. 10.1142/9789814295291_0010 19908360

[51] Pundhir S, Gorodkin J. Differential and coherent processing patterns from small RNAs. Sci Rep. 2015;5:12062 10.1038/srep12062 26166713 PMC4499813

[52] Massenet S. In vivo assembly of eukaryotic signal recognition particle: A still enigmatic process involving the SMN complex. Biochimie. 2019;164:99–104. 10.1016/j.biochi.2019.04.007 30978374

[53] Koziol U, Rauschendorfer T, Zanon Rodríguez L, Krohne G, Brehm K. The unique stem cell sysem of the immortal larva of the human parasite *Echinococcus multilocularis*. Evodevo. 2014;5(1):10 10.1186/2041-9139-5-10 24602211 PMC4015340

[54] Zamanian M, Fraser LM, Agbedanu PN, Harischandra H, Moorhead AR, Day TA, et al Release of Small RNA-containing Exosome-like Vesicles from the Human Filarial Parasite *Brugia malayi*. PLoS Negl Trop Dis. 2015;9(9):1–23. 10.1371/journal.pntd.0004069 26401956 PMC4581865

[55] Raabe C a Tang T-H, Brosius J Rozhdestvensky TS. Biases in small RNA deep sequencing data. Nucleic Acids Res. 2014;42(3):1414–26. 10.1093/nar/gkt1021 24198247 PMC3919602

[56] Wei Z, Batagov AO, Schinelli S, Wang J, Wang Y, El Fatimy R, et al Coding and noncoding landscape of extracellular RNA released by human glioma stem cells. Nat Commun. 2017;8(1):1145 10.1038/s41467-017-01196-x 29074968 PMC5658400

[57] Chiou NT, Kageyama R, Ansel KM. Selective Export into Extracellular Vesicles and Function of tRNA Fragments during T Cell Activation. Cell Rep. 2018;25(12):3356–3370.e4. 10.1016/j.celrep.2018.11.073 30566862 PMC6392044

[58] Liang P, Mao L, Zhang S, Guo X, Liu G, Wang L, et al Identification and molecular characterization of exosome-like vesicles derived from the *Taenia asiatica* adult worm. Acta Trop. 2019;198:105036 10.1016/j.actatropica.2019.05.027 31125559

[59] Britton C, Winter AD, Marks ND, Gu H, McNeilly TN, Gillan V, et al Application of small RNA technology for improved control of parasitic helminths. Vet Parasitol. 2015;212(1–2):47–53. 10.1016/j.vetpar.2015.06.003 26095949 PMC4535316

[60] Guo X, Zheng Y. Expression profiling of circulating miRNAs in mouse serum in response to *Echinococcus multilocularis* infection. Parasitology. 2017;144(8):1079–87. 10.1017/S0031182017000300 28270244

[61] Wang L, Jia X-J, Jiang H-J, Du Y, Yang F, Si S-Y, et al MicroRNAs 185, 96, and 223 Repress Selective High-Density Lipoprotein Cholesterol Uptake through Posttranscriptional Inhibition. Mol Cell Biol. 2013; 33(10):1956–1964. 10.1128/MCB.01580-12 23459944 PMC3647964

[62] Jeon T Il, Esquejo RM, Roqueta-Rivera M, Phelan PE, Moon YA, Govindarajan SS, et al An SREBP-responsive microRNA operon contributes to a regulatory loop for intracellular lipid homeostasis. Cell Metab. 2013;18(1):51–61. 10.1016/j.cmet.2013.06.010 23823476 PMC3740797

[63] Tsai IJ, Zarowiecki M, Holroyd N, Garciarrubio A, Sánchez-Flores A, Brooks KL, et al The genomes of four tapeworm species reveal adaptations to parasitism. Nature. 2013;496(7443):57–63. 10.1038/nature12031 23485966 PMC3964345

[64] Li G, Li J, Li C, Qi H, Dong P, Zheng J, et al MicroRNA-125a-5p contributes to hepatic stellate cell activation through targeting FIH1. Cell Physiol Biochem. 2016;38(4):1544–52. 10.1159/000443095 27074047

[65] Brehm K, Koziol U. *Echinococcus*–Host Interactions at Cellular and Molecular Levels. Advances in Parasitology. 2017;95:147–212. 10.1016/bs.apar.2016.09.001 28131363

[66] Wei Z, Batagov AO, Carter DRF, Krichevsky AM. Fetal Bovine Serum RNA Interferes with the Cell Culture derived Extracellular RNA. Sci Rep. 2016;6:31175 10.1038/srep31175 27503761 PMC4977539

[67] Martinez G. tRNA-derived small RNAs: New players in genome protection against retrotransposons. RNA Biology. 2018;15(2):170–175. 10.1080/15476286.2017.1403000 29120263 PMC5798961

