# Supplementary figures and images for: Extracellular non-coding RNA signatures of the metacestode stage of Echinococcus multilocularis
Source: PLoS Negl Trop Dis. 2020 Nov 30;14(11):e0008890. doi: 10.1371/journal.pntd.0008890 (PMC7728270; doi:10.1371/journal.pntd.0008890)

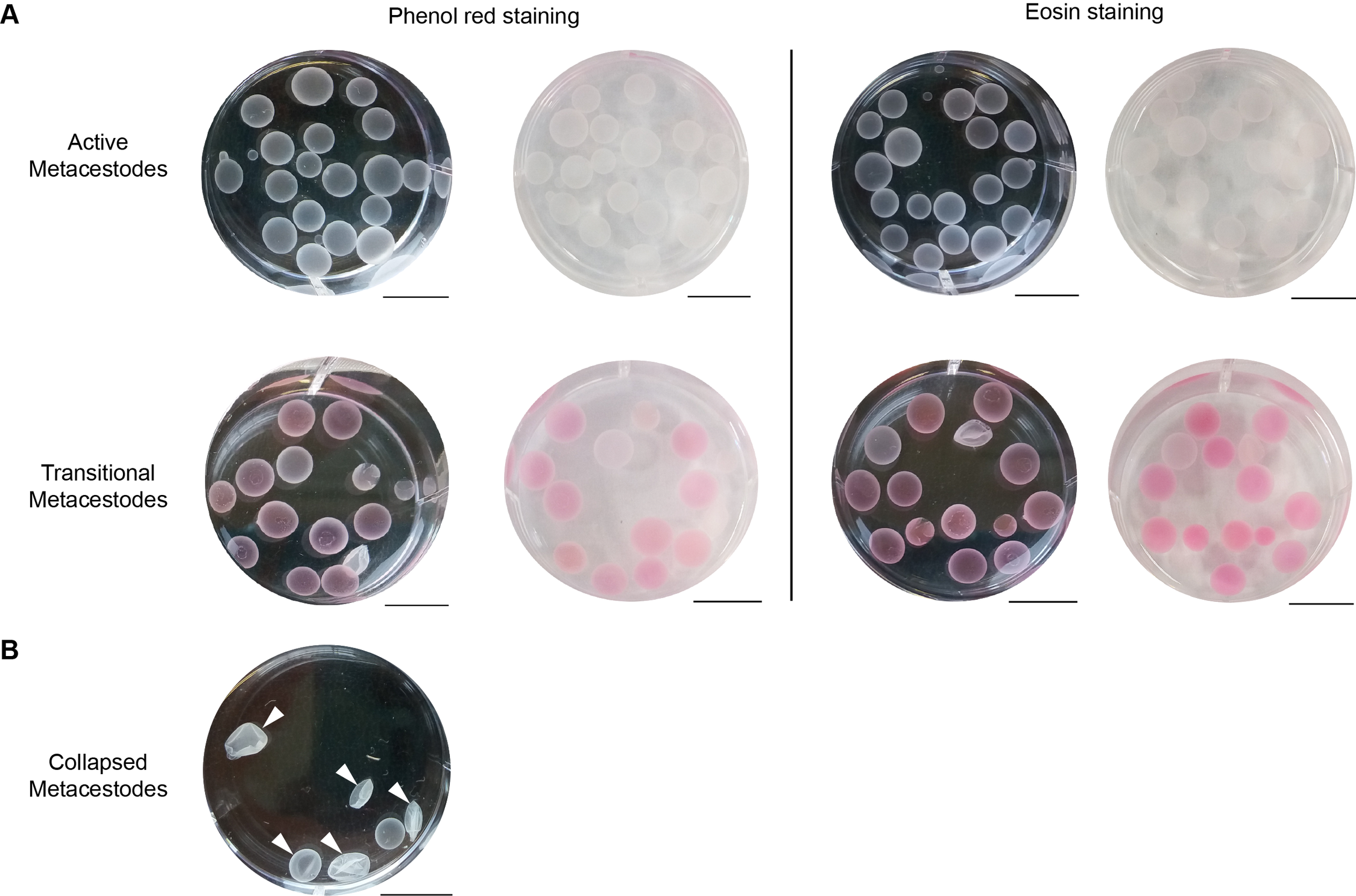

Supplement: S1 Fig — A) Classification of Active and Transitional metacestodes according to phenol red staining (final concentration 0.04 mM) and eosin staining (final concentration 0.02%). B) Collapsed metacestodes with loss of turgency were excluded from cultures. White arrows show collapsed metacestodes. Scale bars indicate 1 cm. (TIF) [file pntd.0008890.s001.tif]

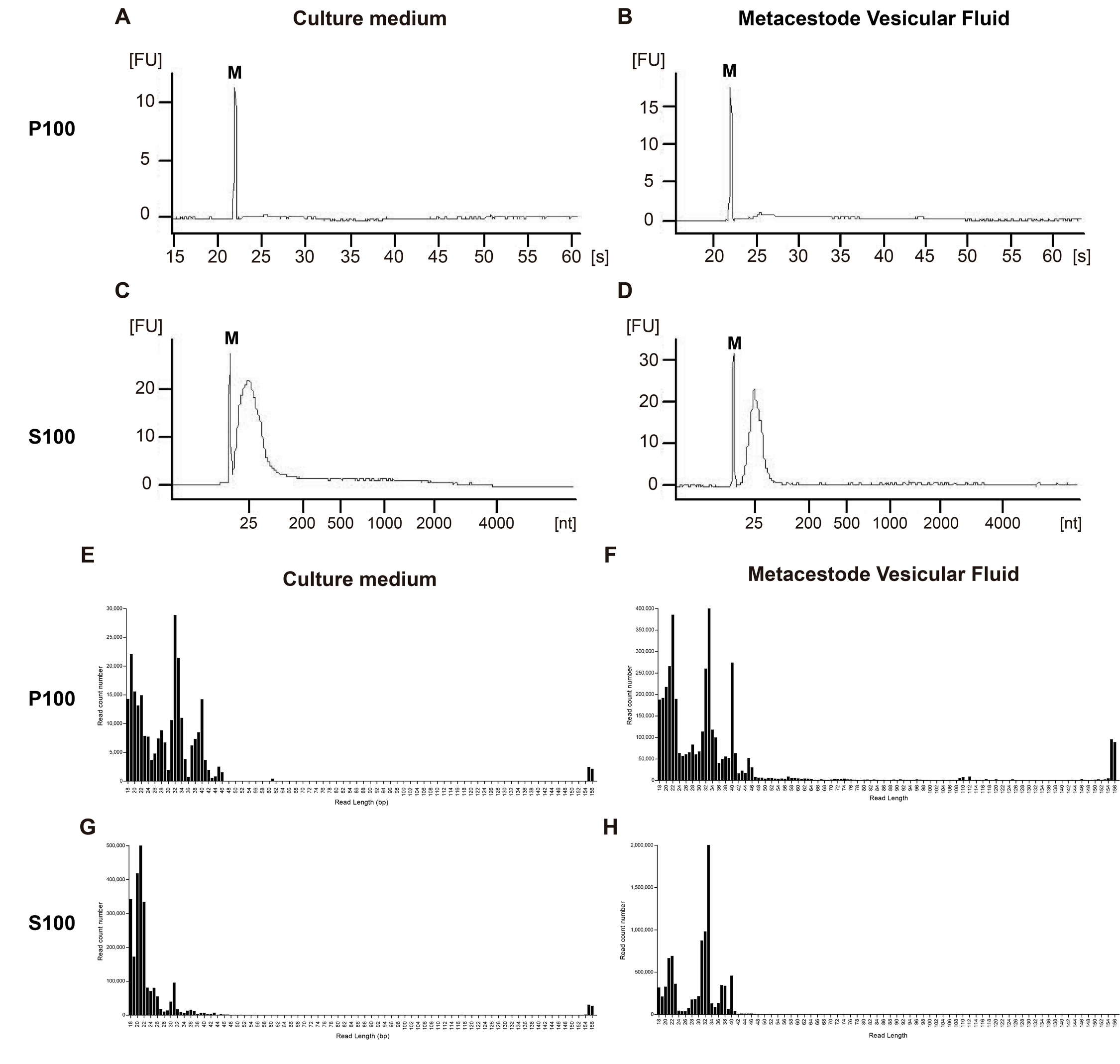

Supplement: S2 Fig — Analysis of the long RNA (> 200 nt) content present in the P100 and S100 fractions of culture medium (A, C) and metacestode vesicular fluid (B, D) of active E. multilocularis metacestodes. M: marker. FU: fluorescence units. Size distribution of reads mapping unambiguously to the E. multilocularis genome detected in the P100 and S100 fractions of culture medium (E, G) and metacestodes vesicular fluid (F, H) of metacestodes. (TIF) [file pntd.0008890.s002.tif]

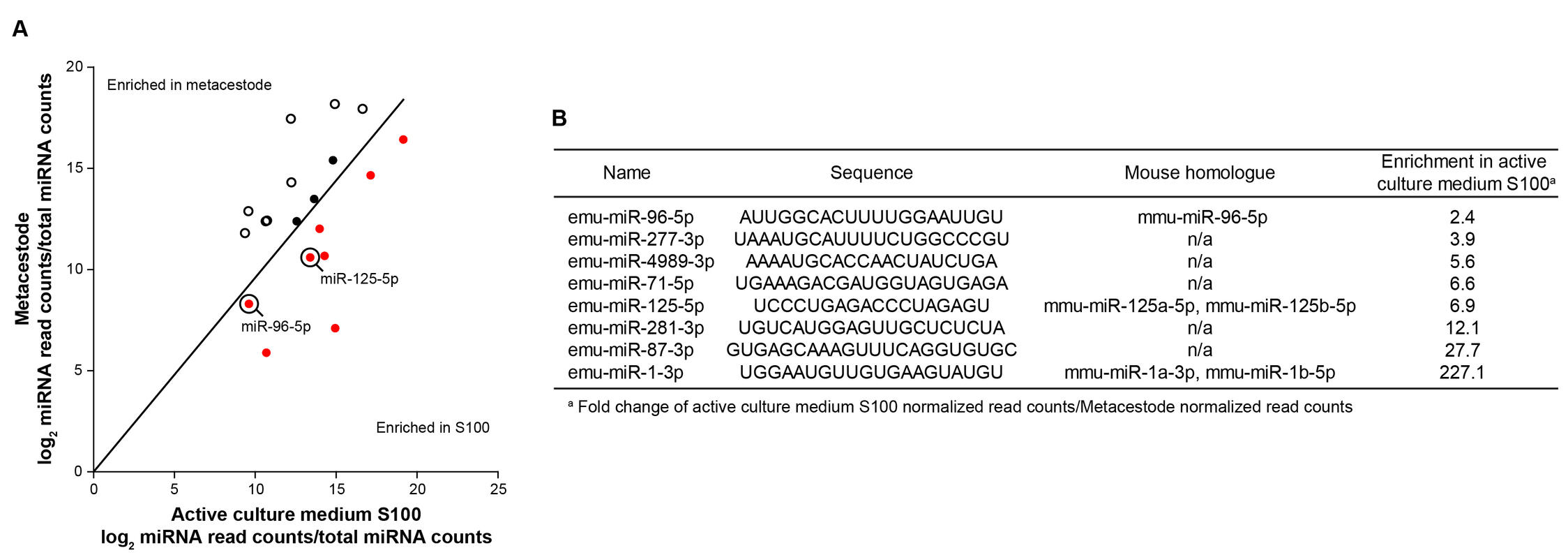

Supplement: S3 Fig — (A) Linear regression of miRNA abundance in fraction S100 of active culture medium and in metacestode tissue. Each dot represents a miRNA. Red dots indicate miRNAs enriched in S100 fraction of active culture medium; hollow dots indicate miRNAs enriched in metacestode tissue; black dots indicate miRNAs with equal abundance. (B) miRNAs enriched in the S100 fraction of active culture medium. The sequence, M. musculus homologous miRNAs and level of enrichment are shown. (TIF) [file pntd.0008890.s003.tif]

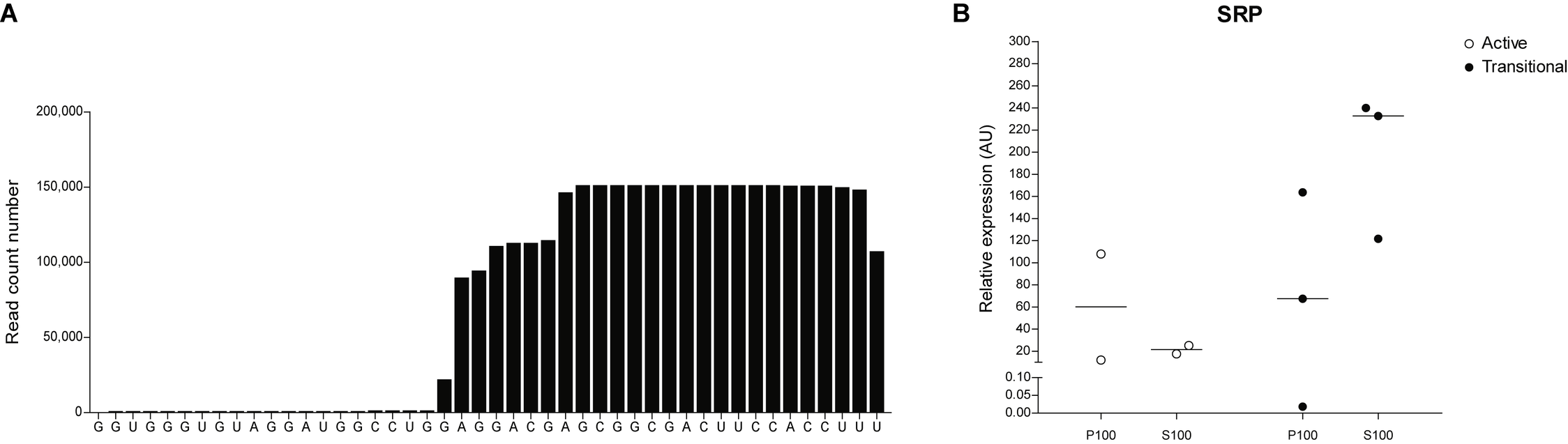

Supplement: S4 Fig — A) Length distribution of reads detected in culture medium of active metacestodes. Sequences corresponding to reads mapping to one specific region (±1 nt) and account for ≥ 50% of total read counts for this gene are shown. Only the 3´-end of the gene is displayed. B) RT-qPCR detection of SRP-derived sequence in P100 and S100 from active and transitional cultures. N = 3 each. Lines indicate the median values. (TIF) [file pntd.0008890.s004.tif]

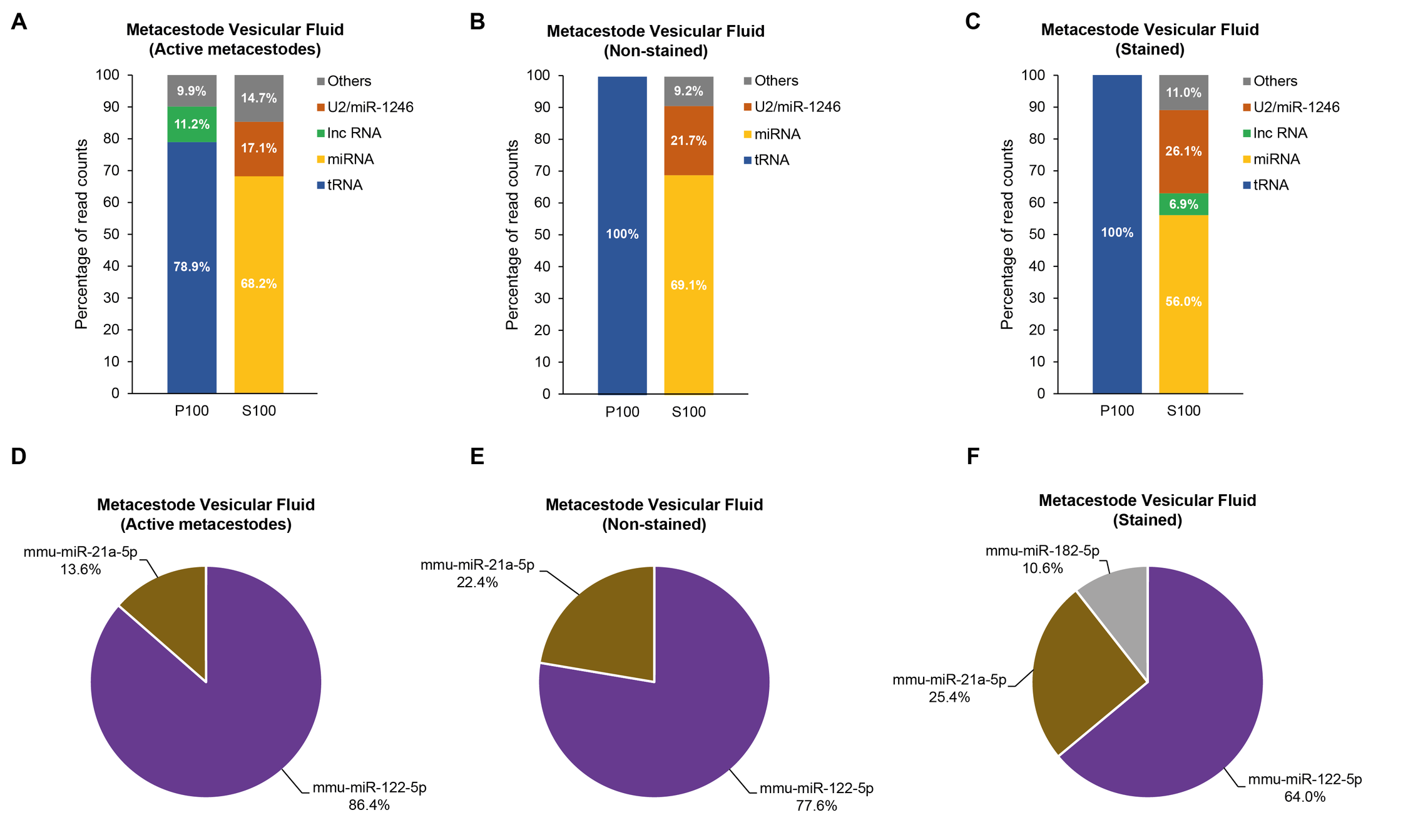

Supplement: S5 Fig — Analysis of the P100 and S100 fractions of metacestode vesicular fluid of E. multilocularis active (A) and transitional (B, C) cultures. Host microRNAs detected in the S100 fraction from metacestode vesicular fluid of active (D) and transitional (E, F) cultures. (TIF) [file pntd.0008890.s005.tif]

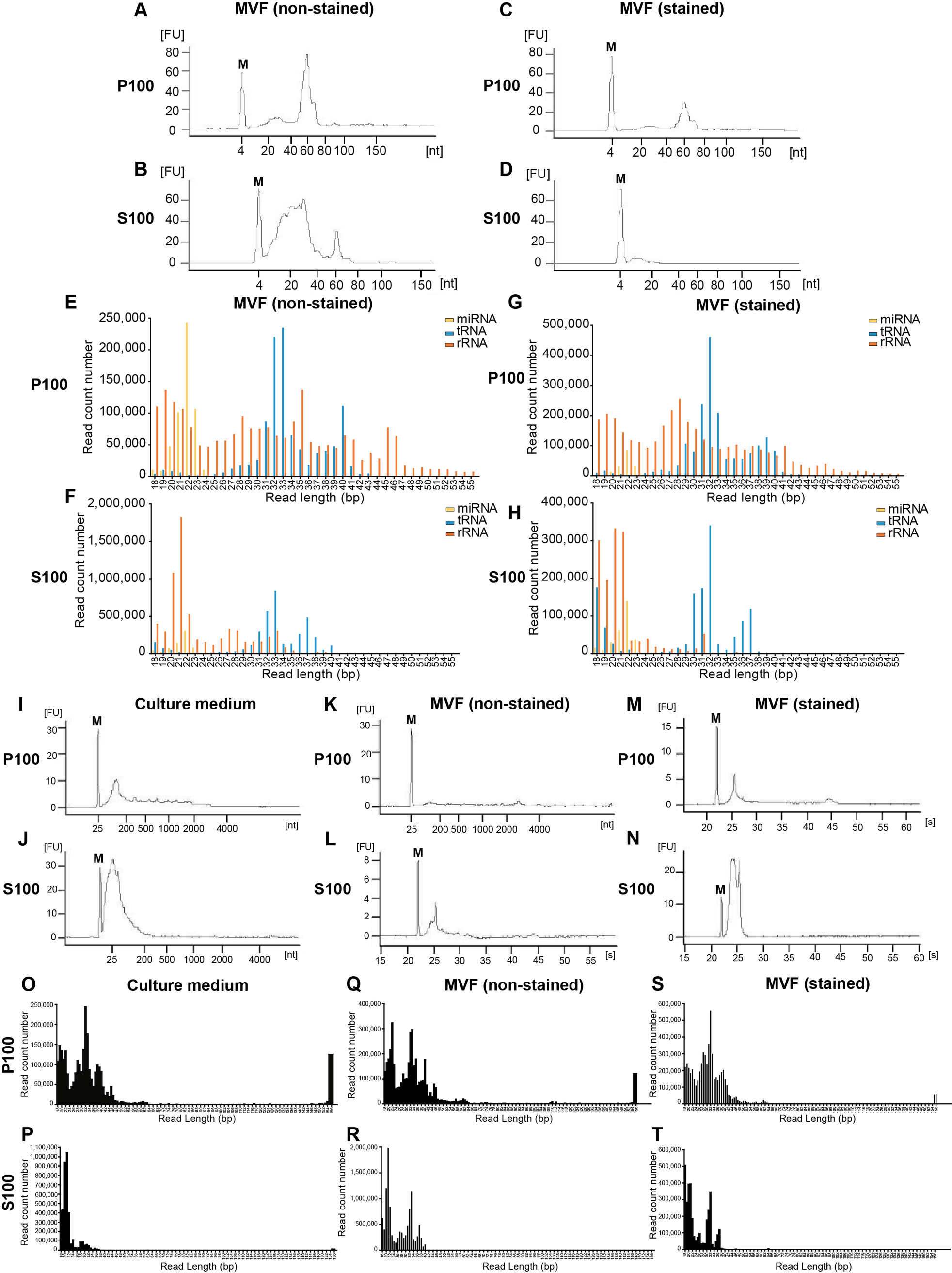

Supplement: S6 Fig — Analysis of the small RNA content (< 200 nt) present in the P100 and S100 fractions of metacestodes vesicular fluid (MVF) from viable (non-stained) (A, B) and senescent (stained) (C, D) metacestodes. Size distribution of the three main RNA biotypes detected in MVF from viable (E, F) and senescent (G, H) metacestodes. Analysis of the large RNA content (> 200 nt) present in the P100 and S100 fractions of culture medium (I, J) and MVF (K-N). M: marker. FU: fluorescence units. General size distribution of reads mapping unambiguously to the E. multilocularis genome detected in culture medium (O, P) and MVF (Q-T). (TIF) [file pntd.0008890.s006.tif]

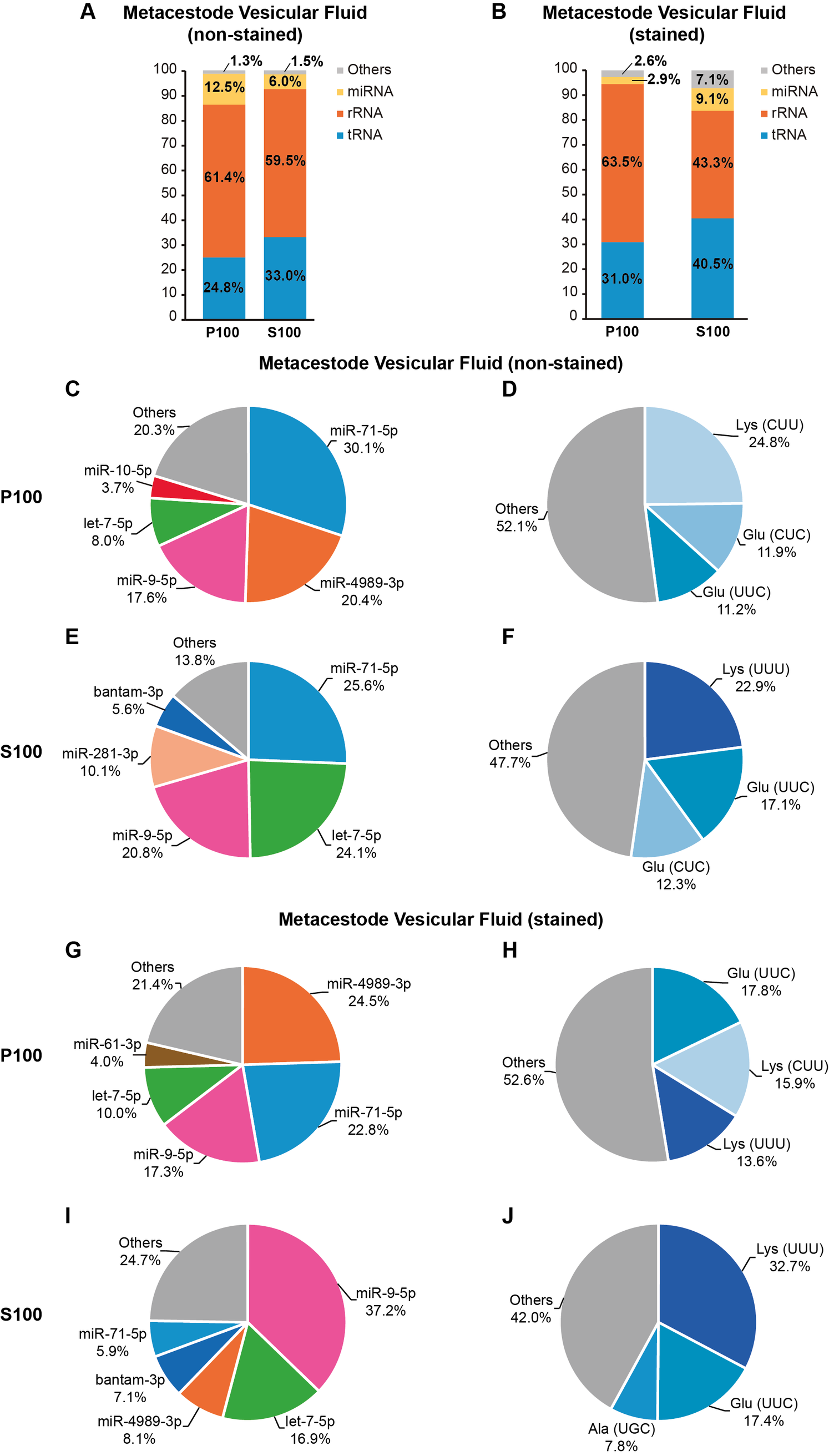

Supplement: S7 Fig — RNA biotypes identified in the P100 and S100 fractions of MVF from viable (non-stained) (A) and senescent (stained) (B) metacestodes. Most abundantly detected miRNAs (C, E, G, I) and tRNA-derived sequences (D, F, H, J) in non-stained and stained MVF. (TIF) [file pntd.0008890.s007.tif]
