# Supplementary material for: Extracellular non-coding RNA signatures of the metacestode stage of Echinococcus multilocularis
Source: PLoS Negl Trop Dis. 2020 Nov 30;14(11):e0008890. doi: 10.1371/journal.pntd.0008890 (PMC7728270; doi:10.1371/journal.pntd.0008890)
Supplement: S1 Table — (DOCX) [file pntd.0008890.s008.docx]

**S1 Table.** Primers used for reverse transcription (RT) and real-time PCR

| **Target (reaction)** | **Sequence (5´- 3´)** | **Annealing temperature** | **Mean Efficiency** |
| --- | --- | --- | --- |
| Stem loop emu-miR-71-5p (SL-RT) | GTCGTATCCAGTGCAGGGTCCGAGGTATTCGCACTGGATACGACTCTCAC | N/A | N/A |
| Stem loop emu-miR-4989-3p (SL-RT) | GTCGTATCCAGTGCAGGGTCCGAGGTATTCGCACTGGATACGACTCAGAT | N/A | N/A |
| Stem loop emu-let-7-5p (SL-RT) | GTCGTATCCAGTGCAGGGTCCGAGGTATTCGCACTGGATACGACAGACAT | N/A | N/A |
| Stem loop hsa-miR-423-5p (SL-RT) | GTCGTATCCAGTGCAGGGTCCGAGGTATTCGCACTGGATACGACAAAGTC | N/A | N/A |
| Stem loop hsa-miR-122-5p (SL-RT) | GTCGTATCCAGTGCAGGGTCCGAGGTATTCGCACTGGATACGACCAAACA | N/A | N/A |
| Stem loop 5p-tRNA^Glu^ (SL-RT)-1 | GTCGTATCCAGTGCAGGGTCCGAGGTATTCGCACTGGATACGACAGCCAG | N/A | N/A |
| Stem loop 5p-tRNA^Glu^ (SL-RT)-2 | GTCGTATCCAGTGCAGGGTCCGAGGTATTCGCACTGGATACGACAACCAG | N/A | N/A |
| Poly T adaptor (poly-A RT) | GCGAGCACAGAATTAATACGACTCACTATAGGTTTTTTTTTTTTVN | N/A | N/A |
| emu-miR-71-5p Forward (PCR) in vitro | GCTGAAAGACGATGGTAGTG | 60ºC | 1.8 |
| emu-miR-71-5p Forward (PCR) in vivo | GCTGAAAGACGATGGTA | 60ºC | 1.8 |
| emu-miR-4989-3p Forward (PCR) | TGGGCAAAATGCACCAACTA | 60ºC | 1.7 |
| emu-let-7-5p Forward (PCR) | TCGCGTGAGGTAGTGTTTCG | 60ºC | 1.7 |
| hsa-miR-423-5p  Forward (PCR) | GTGAGGGGCAGAGAGCGAG | 60ºC | 1.9 |
| hsa-miR-122-5p  Forward (PCR) | TCGCGTGGAGTGTGACAATGGT | 60ºC | 1.8 |
| 5p-tRNA^Glu^ Forward (PCR) | TCCCTGATGGTCTAGCGGT | 60ºC | 1.8 |
| Reverse stem loop (PCR) | CCAGTGCAGGGTCCGAGGT | N/A | N/A |
| 5p-tRNA^Ala^ Forward (PCR) | TCGCGGGGATGTAGCTCAG | 64ºC | 1.9 |
| 5p-tRNA^Gly^ Forward (PCR) | GCGCCGGTGGTTCAGTGGTAG | 64ºC | 1.9 |
| SRP (PCR) | AGGACGAGCGGCGACTTCCACC | 64ºC | 1.9 |
| Reverse poly-A (PCR) | GCGAGCACAGAATTAATACGAC | N/A | N/A |

SL-RT: stem loop reverse transcription

Poly-A RT: poly-A reverse transcription

N/A: Not applicable
