# Supplementary material for: Extracellular non-coding RNA signatures of the metacestode stage of Echinococcus multilocularis
Source: PLoS Negl Trop Dis. 2020 Nov 30;14(11):e0008890. doi: 10.1371/journal.pntd.0008890 (PMC7728270; doi:10.1371/journal.pntd.0008890)
Supplement: S4 Table — (DOCX) [file pntd.0008890.s011.docx]

**S4 Table.** *Echinococcus multilocularis* rRNA-derived small RNAs detected in S100 fractions

| Source | Read counts | Gene ID | Mapping starting position | Read Sequence (%)* | Read length (nt) |
| --- | --- | --- | --- | --- | --- |
| MVF  (Active cultures) | 449,809 | URS000042B970_6211 Echinococcus multilocularis large subunit ribosomal RNA (28S) | 298 | CUGCCGGGUACGACAAUGAAAGACCACUCCC (50.2 %) | 31 |
|  | 265,175 | URS0000C65767_6211  Echinococcus multilocularis Eukaryotic small subunit ribosomal RNA | 553-554 | CGGCGUUGGGGUGGCUGUGCA (38.6 %)  CGGCGUUGGGGUGGCUGUGC (16.0 %) | 21  20 |
| Transitional culture medium | 148,980 | URS000042B970_6211 Echinococcus multilocularis large subunit ribosomal RNA (28S) | 308 | CGACAAUGAAAGACCACUCC (94.8 %) | 20 |
| Non-stained MVF (Transitional cultures) | 3,144,396 | URS0000C65767_6211 Echinococcus multilocularis Eukaryotic small subunit ribosomal RNA | 553-554 | CGGCGUUGGGGUGGCUGUGCA (44. 0%)  CGGCGUUGGGGUGGCUGUGC (26.7 %) | 21  20 |
|  | 685,228 | URS0000C80A48_6211 Echinococcus multilocularis Eukaryotic small subunit ribosomal RNA | 796-797 | CUGCGCCCGCGUGGGUGACGUUGGACGAUGCCG (28.2 %)  UGCGCCCGCGUGGGUGACGUUGGACGAUGCCG (15.8 %)  CUGCGCCCGCGUGGGUGACGUUGGACGAUGCC (8.9 %) | 33  32  32 |
| Stained MVF (Transitional cultures) | 542,338 | URS0000C65767_6211 Echinococcus multilocularis Eukaryotic small subunit ribosomal RNA | 554 | CGGCGUUGGGGUGGCUGUGCA (44.6 %)  CGGCGUUGGGGUGGCUGUGC (34.9 %) | 21  20 |

* Sequences corresponding to reads mapping to one specific region (±1 nt), with similar length (± 3 nt) and accounting for ≥ 50% of total read counts for the corresponding gene are shown.

MVF: Metacestode vesicular fluid.
