# Supplementary material for: Extracellular non-coding RNA signatures of the metacestode stage of Echinococcus multilocularis
Source: PLoS Negl Trop Dis. 2020 Nov 30;14(11):e0008890. doi: 10.1371/journal.pntd.0008890 (PMC7728270; doi:10.1371/journal.pntd.0008890)
Supplement: S6 Table — (DOCX) [file pntd.0008890.s013.docx]

**S6 Table.** General results of small RNA sequencing of ex-RNAs present in culture medium and metacestode vesicular fluid (MVF) of *E. multilocularis* transitional cultures.

|  | **Culture Medium** | | **MVF**  **(non-stained)** | | **MVF**  **(stained)** | |
| --- | --- | --- | --- | --- | --- | --- |
|  | **P100** | **S100** | **P100** | **S100** | **P100** | **S100** |
| **Raw reads** | 15,593,492 | 15,260,310 | 17,457,079 | 30,562,904 | 23,045,300 | 25,691,747 |
| **Pre-processed reads^a^** | 7,234,798 | 5,214,050 | 7,748,605 | 12,099,995 | 13,451,123 | 3,779,028 |
| **Mapped unambiguously to the *E. multilocularis* genome** | 2,893,889  (40.0%) | 3,952,273  (75.8%) | 4,217,784  (54.4%) | 11,177,856  (92.4%) | 5,811,032 (43.2%) | 3,230,122 (85.5%) |
| **Mapped unambiguously to the *Mus musculus* genome** | 150,233  (2.1%) | 535,973  (10.3%) | 2,074  (0.03%) | 4,798  (0.04%) | 1,163 (0.01%) | 8,607 (0.2%) |
| **Mapped ambiguously to both genomes** | 0  (0.0%) | 16,315  (0.3%) | 190,249  (2.5%) | 110,652  (0.9%) | 7,470 (0.06%) | 47,052 (1.2%) |
| ***E. multilocularis* miRNAs^b^** | 171,304 | 2,532,932 | 561,944 | 707,381 | 189,101 | 314,572 |
| **Vertebrate miRNAs^b^** | 0 | 275,064 | 0 | 4,340 | 0 | 5,890 |
| ***E. multilocularis* tRNAs** | 700,986 | 597,083 | 1,047,879 | 3,685,247 | 1,800,698 | 1,309,348 |
| **Vertebrate tRNAs** | 130,225 | 11,470 | 2,074 | 0 | 1,163 | 0 |
| ***E. multilocularis* rRNAs** | 1,961,259 | 739,693 | 2,589,573 | 6,651,257 | 3,692,274 | 1,397,123 |
| **Vertebrate rRNAs** | 1,929 | 392 | 0 | 0 | 0 | 0 |

^a^ Pre-processed reads includes those with: no adaptors, PHRED > 20, length ≥ 18 bp, ≥350 counts.

^b^ miRDeep prediction.
